# Patient-Derived Xenografts of High-Grade Serous Ovarian Cancer Subtype as a Powerful Tool in Pre-Clinical Research

**DOI:** 10.3390/cancers13246288

**Published:** 2021-12-15

**Authors:** Magdalena Cybula, Lin Wang, Luyao Wang, Ana Luiza Drumond-Bock, Katherine M. Moxley, Doris M. Benbrook, Camille Gunderson-Jackson, Maria J. Ruiz-Echevarria, Resham Bhattacharya, Priyabrata Mukherjee, Magdalena Bieniasz

**Affiliations:** 1 Aging and Metabolism Program, Oklahoma Medical Research Foundation, Oklahoma City, OK 73104, USA; Magdalena-Cybula@omrf.org (M.C.); Lin-Wang@omrf.org (L.W.); Summer-Wang@omrf.org (L.W.); AnaLuiza-Bock@omrf.org (A.L.D.-B.); 2 Division of Gynecologic Oncology, Department of Obstetrics and Gynecology, Stephenson Cancer Center, University of Oklahoma Health Science Center, Oklahoma City, OK 73104, USA; Katherine-Moxley@ouhsc.edu (K.M.M.); Doris-Benbrook@ouhsc.edu (D.M.B.); Camille-jackson@ouhsc.edu (C.G.-J.); Resham-Bhattacharya@ouhsc.edu (R.B.); Priyabrata-Mukherjee@ouhsc.edu (P.M.); 3 Department of Pathology, Department of Medicine, Stephenson Cancer Center, University of Oklahoma Health Science Center, Oklahoma City, OK 73104, USA; Maria-RuizEchevarria@ouhsc.edu

**Keywords:** ovarian cancer, patient-derived xenograft, tumor model

## Abstract

**Simple Summary:**

Patient-derived xenografts (PDXs) have gained popularity as a model system in anti-cancer drug development. PDXs are established by the transfer of patient tumors directly into mice without prior in vitro manipulation, assuming that these models closely resemble patient tumors. However, recent reports have shown that tumor evolution can result in genomic alterations of PDXs, emphasizing the need to assess the extent of genetic drift in PDX models. To address this need, we developed a method to interrogate genetic drift in a panel of ovarian cancer PDXs using SNP genotyping. We demonstrated that PDX models retain molecular and histological characteristics of the original patients’ tumors even following multiple passages in mice. Further, we showed that these models faithfully recapitulate the therapeutic response of their corresponding patients. Overall, validated patient-derived models of ovarian cancer are valuable tools to facilitate translation of new therapies from pre-clinical studies to patients.

**Abstract:**

(1) Background. PDX models have become the preferred tool in research laboratories seeking to improve development and pre-clinical testing of new drugs. PDXs have been shown to capture the cellular and molecular characteristics of human tumors better than simpler cell line-based models. More recently, however, hints that PDXs may change their characteristics over time have begun to emerge, emphasizing the need for comprehensive analysis of PDX evolution. (2) Methods. We established a panel of high-grade serous ovarian carcinoma (HGSOC) PDXs and developed and validated a 300-SNP signature that can be successfully utilized to assess genetic drift across PDX passages and detect PDX contamination with lymphoproliferative tissues. In addition, we performed a detailed histological characterization and functional assessment of multiple PDX passages. (3) Results. Our data show that the PDXs remain largely stable throughout propagation, with marginal genetic drift at the time of PDX initiation and adaptation to mouse host. Importantly, our PDX lines retained the major histological characteristics of the original patients’ tumors even after multiple passages in mice, demonstrating a strong concordance with the clinical responses of their corresponding patients. (4) Conclusions. Our data underline the value of defined HGSOC PDXs as a pre-clinical tumor model.

## 1. Introduction

High-grade serous ovarian carcinoma is the most aggressive ovarian cancer subtype, and accounts for two-thirds of all ovarian cancer deaths, making it by far the most lethal gynecological malignancy [1,2]. As precision medicine opens up promising new treatment strategies for ovarian cancer, the translation of research findings into new therapies is still an enormous barrier to progress. The collective data demonstrate that ~85% of anti-cancer drugs entering clinical trials fail to demonstrate sufficient safety or efficacy to gain regulatory approval [3]. This high failure rate reflects the weak understanding of the complexity of human cancer and the limitations of existing pre-clinical tumor models [3,4]. Long-established cancer cell lines and cell line-based xenograft models have long been the workhorse of cancer research; however, the marked differences between in vitro cell culture conditions and the in vivo tumor environment raise concerns that these models lack proper representation of actual human tumors [4,5]. In addition, a comprehensive study revealed profound molecular differences between the common cell lines used to model high-grade serous ovarian carcinoma and patient tumor specimens of the same cancer subtype, which highlights the serious limitations of these models’ predictive value in terms of clinical efficacy [1]. Hence, there is a need for tumor models that better replicate the diversity and heterogeneity of defined ovarian cancer subtypes.

In last decade, significant efforts have been made to expand development of patient-derived xenograft (PDX) models from a wide spectrum of human cancers in order to provide improvement over cell line-based models that are poor surrogates for actual disease [5,6,7]. A number of studies have reported that PDX models faithfully recapitulate key features of the original tumors [7,8,9,10,11]. More recently, however, hints that PDXs may change their characteristics over time have begun to emerge, emphasizing the need for comprehensive analysis of PDX authenticity and tumor evolution throughout propagation [12,13]. A commonly-used method for verifying the authenticity of human cell lines and tissues is short tandem repeat (STR) profiling. The limitation of this method, however, is that cell lines with defects in their DNA repair system can alter their STR profile upon long-term passaging, leading to false authentication results [14]. This poses a limitation for the use of STR profiling in the validation of tissues characterized by genomic instability, including ovarian cancer. To circumvent this issue, various SNP-based methods for verification of tissue authenticity have been developed, demonstrating that a selected set of SNPs is able to correctly identify any cell line/tissue regardless of passage [15,16]. In this study, we established a panel of ovarian PDX models and developed a robust method to assess genetic drift across PDX passages, detect PDX contamination with lymphoproliferative tissues, and perform PDX authentication using high-throughput SNP genotyping. In addition, we performed a detailed histological and molecular characterization and functional assessment of serial PDX passages over multiple generations. In addition, we discuss the requirements for a rigorous strategy to prevent contamination of PDX lines with lymphoproliferative neoplasms, which is a common problem accompanying PDX generation using immunocompromised mouse hosts. To evaluate the predictive value of PDX models for clinical outcomes, we compared the therapeutic responses of PDXs to the treatment responses of their corresponding patients. Finally, in order to expand the use of PDX lines for in vivo orthotopic tumor modeling, we optimized efficient methods to luciferize PDXs, enabling non-invasive bioluminescence imaging in living mice.

## 2. Materials and Methods

### 2.1. Patients and Tissue Samples

Tumors from ovarian cancer patients were obtained via core needle biopsy or surgical resection, following informed consent. Patient tumor samples were collected at the initial diagnosis prior any treatment (chemotherapy-naïve tumors). Collected tissues were immediately placed into a sterile tube containing ice-chilled Dulbecco’s Modified Eagle’s Medium (DMEM) and transported to the Patient-Derived Xenograft and Preclinical Therapeutics (PDX-PCT) Core at OMRF, then immediately processed for engraftment and/or cryopreservation. We followed rigorous protocols to process human tumor specimens within 2 h of receiving the tissue from operating room. Patients’ ovarian tumors were processed by removal of necrotic tissue and fat, followed by cutting the viable tumor tissue with a scalpel into smaller fragments (~4 mm × 2 mm in size) under aseptic sterile conditions. From each collected human tumor specimen, we obtained ~5–20 viable tumor fragments after processing. One tumor fragment was fixed in 10% buffered formalin and embedded into paraffin blocks, while the remaining tumor fragments were either immediately implanted into mice or cryopreserved in freezing medium (95% FBS, 5% DMSO) [17]. The PDX-PCT core collected only de-identified tissue samples and clinical–pathologic data from enrolled patients diagnosed and treated at the Stephenson Cancer Center at the University of Oklahoma. The PDX tumor model collection is stored at the PDX-PCT core facility at OMRF.

### 2.2. PDX Models

For animal studies, immunodeficient mouse strains obtained from the Jackson Laboratory were used, including NOD/scid mice (#001303—NOD.Cg-Prkdcscid/J), NRG mice (#007799—NOD.Cg-Rag1tm1Mom Il2rgtm1Wjl/SzJ) and NSG mice (#005557—NOD.Cg-Prkdcscid Il2rgtm1Wjl/SzJ) [18,19]. In this study, we used female mice due to the nature of ovarian cancer, which affects only women. For PDX model development, six week-old female mice (*n* = 5 per tumor sample) were subcutaneously (SQ) implanted in the dorsal flank with a patient tumor fragment. Prior to implantation, patient tumors were processed and cut into small viable tumor fragments (4 mm × 2 mm in size) that were either immediately implanted into mice as “fresh tumors”, or frozen and then implanted into mice at later time as “frozen/thawed” tumors [17]. The mice were monitored bi-weekly for tumor development. Tumor growth was evaluated by caliper measurements, and tumor volume was calculated using the formula ½ (Length × Width^2^). A mouse was sacrificed once the tumor volume reached 1000 mm^3^, it exceeded 12 months of age, or it showed symptoms of health decline. Harvested tumors were processed and cryopreserved and/or serially passaged by SQ implantation into new recipient mice to establish further PDX generations. For experiments including PDX development from cell suspension, animals were SQ implanted with 1 × 10^6^ of cells suspended in 50% matrigel in HBSS. All animals were monitored weekly for body weight, development and progression of ovarian tumors, and any symptoms of physical distress or illness.

### 2.3. Morphologic and Immunohistochemical (IHC) Analyses of PDX Tumors

Human tumors or PDX lines were analyzed by IHC for expression of HGSOC markers and to detect contamination with lymphoma. Harvested tumors were fixed in 10% neutral buffered formalin, paraffin-embedded, and hematoxylin–eosin (H&E) stained, according to our standard protocols [20,21]. Tumor tissues were stained with the following antibodies: anti-human cytokeratin (1:400, DAKO #Z0622), PAX8 (1:1000, Abcam, #ab189249), WT1 (1:1250, Cell Signaling, #83535), CD45 (1:1200, Cell Signaling, #13917S), CD20 (1:1000, Abcam, #ab64088) and CD3 (1:1000, Abcam, #ab11089). Staining was visualized by 3,3-diaminobenzidine (DAB), with hematoxylin as a counter-stain. Staining was visualized by 3,3-diaminobenzidine (DAB), with hematoxylin as a counter-stain. Slides were imaged on a Zeiss AxioObserver.Z1 microscope using ZEN 2.3 pro imaging software.

### 2.4. Genome-Wide Genotyping of SNPs in Multiple Passages of PDX Lines

Genomic DNA was isolated from primary high-grade serous ovarian tumors and their corresponding PDX lines using the DNeasy Blood & Tissue Kit (Qiagen, Hilden, Germany). The samples were processed and genotyped using Infinium Exome-24 v1.1 Kit (cat. no. 20015246, Illumina, San Diego, CA, USA) within Clinical Genomics Center at OMRF. Infinium Exome V1.1 arrays deliver unparalleled coverage of putative functional exonic variants consisting of >250,000 markers representing diverse human populations. The arrays were processed using the Infinium HTS protocol. Briefly, using propriety reagents and equipment specifically designed for Infinium assays, 200 ng of genomic DNA was amplified, fragmented, precipitated, resuspended, hybridized to the oligos lining the beadchip, extended by a single base, stained, and scanned on an iScan high-resolution optical imaging system. The DNA was denatured and isothermally amplified by whole-genome amplification, hybridized to the arrays and scanned. The dual-color intensity data were normalized, clustered, and analyzed using Illumina’s GenomeStudio software package.

### 2.5. Isolation of Human Tumor Cells from Ovarian Cancer PDXs

A Tumor Dissociation Kit (Miltenyi Biotec, #130-095-929, Bergisch Gladbach, Germany) was used to deplete mouse cells from PDX tumors and isolate pure populations of human tumor cells. Briefly, PDX tumor tissue was harvested and processed in sterile conditions by removing fat and fibrous and necrotic tissues and preserving the viable tumor material. Up to 1 g of viable PDX tumor tissue was used to dissociate the tissue into single cell suspension by the incubation of fragmented PDXs with a mixture of digestion enzymes for 1 h at 37 °C. Following incubation with digestion enzymes, the sample material was collected by short centrifugation, resuspended in RPMI 1640 medium and passed through a 70 μm single-cell strainer. Next, the mixture of human and mouse cells isolated from PDX tumor was centrifuged, resuspended in fresh medium and counted. In order to preserve only human tumor cells and deplete mouse cells from the PDX cells mixture, a Mouse Cell Depletion Kit (Miltenyi Biotec, #130-104-694, Bergisch Gladbach, Germany) was used. Briefly, mouse cells were labeled with a cocktail of mouse-specific antibodies conjugated with MACS MicroBeads via 15 min incubation at 4 °C. Then, the cells (up to 2 × 10^7^) were passed through the MACS LS Column in the magnetic field of a MidiMACS Separator (Miltenyi Biotec, #130-042-302, Bergisch Gladbach, Germany), and the negative fraction containing human tumor cells was collected. The magnetically labeled mouse tumor stromal cells were retained within the MACS LS Column and discarded. 

### 2.6. Staining of PDX Cell Populations for Flow Cytometry Analysis

Flow cytometry analysis was performed to assess the cellular composition of individual PDX lines and evaluate the purity and yield of human tumor cells isolated from each PDX tumor. Cells obtained from the PDX dissociation procedure were suspended in 80 μL of MACS buffer (PBS pH 7.2, 0.5% BSA, 2mM EDTA) and plated at a density of 0.5 × 10^6^ cells per well in 96-well plate. Next, 20 μL of human FcR blocking reagent (Miltenyi Biotec, #130-059-901) was added to the cells and the cells were incubated for 5 min at 4 °C. Following the incubation and washing of cells, a human-specific antibody, CD326 (EpCAM)-PE (Miltenyi Biotec, #130-113-264, dilution 1:11) for detection of carcinoma cells and/or a Labeling Check Reagent-APC (Miltenyi Biotec, #130-122-219, dilution 1:4) for the detection of mouse cells labeled with MACS MicroBeads, was added to the respective wells and incubated for 10 min at 4 °C. Finally, the cells were washed, resuspended in 300 μL of MACS buffer, and processed using a BD FacsCelesta analyzer (BD Biosciences, San Jose, CA, USA) according to manufacturer’s protocol. The flow cytometry data was analyzed using FlowJo software v10 [22].

### 2.7. Primary Tumor Cell Transduction with LUC-ZsGreen Bicistronic Lentiviruses

To generate luciferized PDX lines, pure populations of human tumor cells isolated from selected PDXs were transduced with concentrated lentiviruses from Addgene (pHIV-LUC-ZsGreen, #39196; pMDLg/pRRE, #12251; pRSV-Rev, #12253 and pCMV-VSV-G, #8454). Production and titering of concentrated lentiviruses was performed according to standard protocols, as described previously [17,20,23]. Briefly, PDX tumor tissue was dissociated into single cell suspension and pure populations of human tumor cells were isolated by magnetic cell sorting. Human tumor cells were cultured for a short term (<7 days) and infected with lentiviruses containing LUC-ZsGreen construct. Five days after lentiviral transduction, the cells were sterile sorted for ZsGreen expression using the FacsAria at the Flow Cytometry Core Facility at OMRF. The sterile sorted cells expressing LUC-ZsGreen construct were immediately implanted into mice to generate luciferized PDX models.

### 2.8. Animal Studies

For the evaluation of chemotherapy response in vivo, six week-old female mice were SQ implanted with a fragment of selected PDX. Then, animals with established tumors of ~200 mm^3^ volume were randomized and treated with cisplatin and/or paclitaxel. Cisplatin and paclitaxel were purchased from the University of Oklahoma Pharmacy and diluted to the desired concentrations in saline or PBS, respectively. For luciferized PDX development, animals were SQ implanted with ZsGreen-LUC positive cells following sterile sorting. The number of implanted tumor cells ranged from 10^5^ to 10^6^ per animal, and was associated with lentiviral transduction efficiency. The growth rate of Luciferized PDXs was monitored weekly by IVIS bioluminescence imaging (Xenogen IVIS, Xenogen, Alameda, CA, USA), coupled to the Living Image 4.5.2 software. Before imaging, mice received intraperitoneal (IP) injections of 150 mg/kg luciferin (Gold Biotechnology, St.Louis, MO, USA). All animals were monitored weekly for body weight, development and progression of ovarian tumors, and any symptoms of physical distress or illness.

### 2.9. Statistical Analysis

Statistical analysis of patient characteristics, PDX data, and in vitro or in vivo assays was done using Fisher’s exact test, unpaired t-test or Dunnett’s test, whenever applicable. Fisher’s exact test was used to assess whether tumor take rate differed with respect to disease characteristics of the patient or type of mouse implanted. The differences in PDX tumor growth kinetics in vivo were assessed using analysis of variance (ANOVA) followed by Tukey’s multiple comparisons test; *p* < 0.05 was considered significant. Statistical analysis was performed using GraphPad Prism 6.0 Software San Diego, CA, USA).

## 3. Results

### 3.1. Establishment of Patient-Derived Xenografts Representing HGSOC Subtype

Between May 2015 and July 2019, a total of 43 chemotherapy-naïve tumor samples were collected from patients undergoing debulking surgery for high-grade serous ovarian cancer. The patient population was represented by women (average age 62.2 years old) with advanced-stage ovarian cancer (98% of women with stage IIIC-IVB and 2% of women with stage IIC) (Table 1 and Supplementary Table S1).

After receiving a tumor sample, we either immediately processed and implanted the tissue as “fresh” tumor fragments into mice, or cryopreserved viable tumor fragments that were implanted later as “frozen/thawed” tumors. For primary tumor implantation and PDX model propagation, we used three immunocompromised mouse strains, NOD/scid, NSG or NRG [18,19,24]. NOD/scid mice are characterized by the absence of lymphocytes B and T as well as reduced macrophage and natural killer (NK) cell function. NSG and NRG mouse strains show slightly greater severity of immunodeficiency as a result of a loss of lymphocytes (B and T), macrophages and NK cells [18,19,24]. We chose the subcutaneous (SQ) route of tumor inoculation and implanted 2 mm × 4 mm tumor fragments into the flank of the recipient mouse. Subcutaneously-grown ovarian PDXs allow accurate tumor volume monitoring and measurement, and have been shown to represent the original human tumor features very well [7].

We successfully engrafted 33 primary ovarian tumor specimens, resulting in a tumor take rate of 77% (Table 1). Successful engraftment was defined as generation of a PDX tumor that had been grown in vivo for at least two passages. We analyzed the clinicopathological features of primary tumors as well as engraftment methods to identify the factors affecting PDX establishment success rates. The results demonstrated that tumor engraftment was not dependent on patient age, tumor stage, response to platinum drugs or overall survival (OS). However, we found that successful engraftment was significantly associated with early tumor recurrence (within 12 months since diagnosis, Table 1). A side-by-side comparison of the engraftment efficiency of fresh tumor specimens and cryopreserved tumor tissues demonstrated 80% and 65% take rates, respectively (Table 1). Engraftment rate in highly immunodeficient NSG or NRG mice was slightly higher (81%) when compared to engraftment rate in less immunodeficient NOD/scid mice (71%, Table 1). The method of tumor preservation (fresh or frozen/thawed) or mouse strain (NSG/NRG or NOD/scid) did not significantly predict successful engraftment. Out of 33 successfully engrafted PDX models, we further expanded through multiple rounds of serial transplantation 17 PDX lines characterized by the growth kinetics suitable for robust in vivo experiments. The remaining 16 PDXs were expanded for around two passages, however due to very slow growth kinetics, we cryopreserved these models without further characterization (Supplementary Table S2).

### 3.2. Lymphoma Transformation in PDX Tumor Models

**Figure 1:**
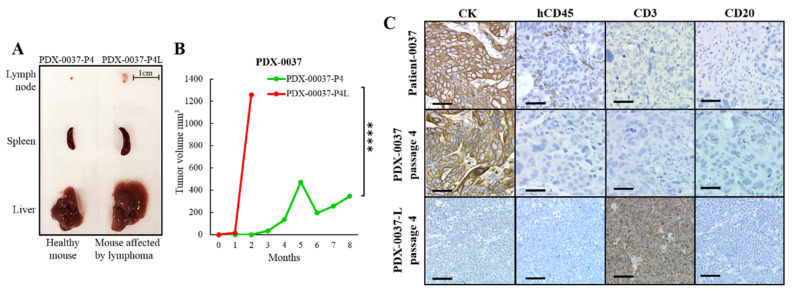
Lymphoma transformation in PDX tumor models. (**A**) Representative photographs of organs collected from a mouse affected by lymphoma vs. healthy control. Macroscopic analysis revealed splenomegaly and enlarged liver and lymph nodes. (**B**) Graph represents tumor growth rate of PDX-0037 with and without lymphoma contamination in NOD/scid mice (one-way ANOVA followed by Tukey’s multiple comparisons test, **** = *p* < 0.0001). (**C**) Representative sections of lymphoma-contaminated tumor model (PDX-0037-P4L) compared with original patient tumor and the non-affected PDX model (PDX-0037-P4); P4 indicates passage number, L indicates individual animal bearing lymphoma-contaminated PDX. Tumor sections were evaluated by IHC for CK, hCD45, CD3 and CD20 expression. Scale bars represents 50 μm. For additional information, see Supplementary Figure S1.

We established a panel of ovarian PDXs by subcutaneous implantation of surgical tumor specimens into immunocompromised mice. We occasionally observed that selected PDX passages in individual animals demonstrated atypical growth kinetics reflected as rapid tumor growth generating soft, flat tumor masses. Mice bearing those atypical tumor grafts showed signs of distress including hunched posture, decreased activity, and/or unexpected death. At necropsy, macroscopic analysis revealed features of lymphoma, including splenomegaly and enlarged liver and lymph nodes (Figure 1A, Supplementary Figure S1). Lymphoma development was apparent in various PDX passages (P2–P5) in individual animals. We discarded all PDX tissues contaminated with lymphoma.

The proof-of-concept experiment revealed that lymphoma-contaminated tumorgrafts grew significantly faster than their corresponding PDX lines maintaining the ovarian carcinoma histology (Figure 1B and Supplementary Figure S1B–F). To verify the species of origin of the lymphoproliferative lesions in PDX-bearing mice, we performed immunohistochemical (IHC) evaluation of the atypical tumorgrafts. Analysis of hematoxylin- and eosin (H&E)-stained tissues revealed densely packed small mononuclear cells with low cytoplasm which had no resemblance to the matched patient’s tumor and did not resemble carcinoma in general (Figure 1C and Supplementary Figure S1G–I). The lymphoma samples were negative for the epithelial marker pan-cytokeratin (CK), indicating a non-epithelial origin, and negative for human specific leukocyte marker CD45, indicating a non-human origin. Further analysis revealed that the lymphomas were reactive with antibodies recognizing a CD3 antigen, but not a CD20 antigen (CD20−/CD3+), which are markers of the T-cell lineage phenotype [25]. These data indicate that the lymphomas developed from mouse pre-T cells, which is consistent with the observation that aging immunodeficient mouse strains are susceptible to spontaneous murine lymphomas (Figure 1C and Supplementary Figure S1G–I) [26,27]. In summary, these data reinforce the requirement of a rigorous strategy to prevent unrecognized lympoproliferations in PDX-bearing mice. Thus, our routine protocols include thorough testing for human/mouse lymphocytic markers in order to eliminate lymphoma-contaminated xenografts from the PDX repository. In addition, we developed an SNP-based PDX authentication method, enabling identification and differentiation of lymphoma-contaminated PDX passages from uncontaminated ones (see “Analysis of the genomic fidelity and stability of PDX models”).

### 3.3. Analysis of HGSOC PDX Tumor Growth Rates

**Figure 2:**
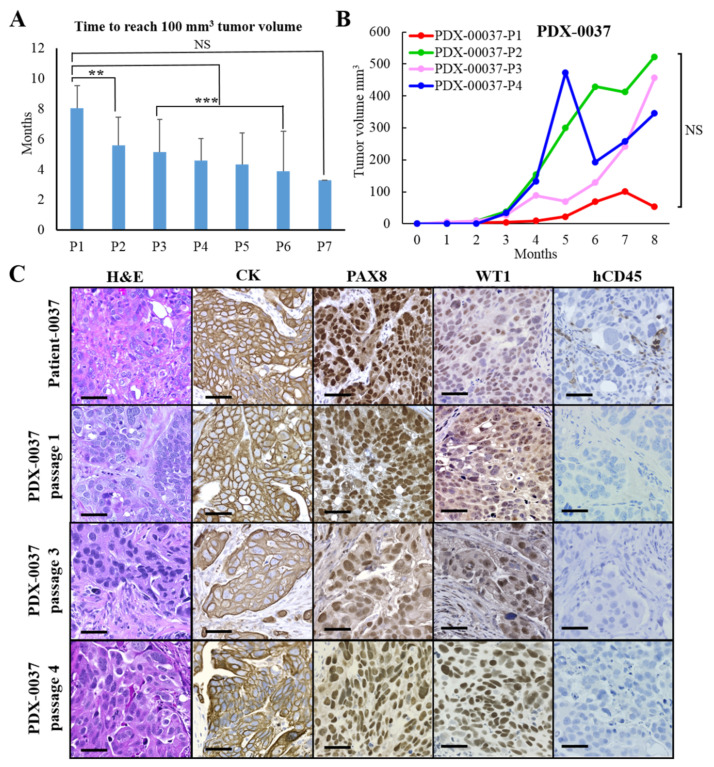
Analysis of tumor growth rates and IHC characterization of PDXs. (**A**) Analysis of tumor growth rates of 17 PDX models. Graph represents an average latency time of each PDX passage to develop a tumor (~100 mm^3^ volume) from the time of implantation. Data are represented as mean ± SEM, illustrating differences between latency time of P1 vs. other passages (one-way ANOVA followed by Tukey’s multiple comparisons test, ** = *p* < 0.01, *** = *p* < 0.001). (**B**) Graph shows growth rates of individual passages of a representative PDX model (one-way ANOVA followed by Tukey’s multiple comparisons test, NS = not significant). (**C**) PDX tumor sections were H&E stained and evaluated by IHC for HGSOC subtype markers (CK, PAX8 and WT1) and the presence of human immune cells (hCD45). Scale bars: 50 μm. For additional information, see Supplementary Figures S2 and S3.

We estimated the tumor growth rates of 17 PDX models across several in vivo passages (up to seven passages). The latency time to develop a clinically apparent disease (tumor volume of ~100 mm^3^) from the time of initial implantation varied from 4 to 10 months for passage 1. We consistently observed that the time required to developed tumors tended to decrease with serial PDX passage (Figure 2A and Supplementary Figure S2). When analyzing individual PDX lines, 11 out of 17 PDXs (65%) showed a statistically significant increase in tumor growth rate as the tumor passage increased, reflected as decreased latency time for each passage to reach tumor size of 100 mm^3^ (Figure 2A and Supplementary Figure S2C). The difference in tumor growth rate between different PDX passages was mostly attributed to the significantly slower tumor growth rate of the initial human-to-mouse passage referred to as passage 1 (P1) vs. later PDX passages (Figure 2A,B).

### 3.4. Immunohistochemical Characterization of PDX Models

We performed a histological and IHC comparison of the original patient tumors and their derivative PDX models. We passaged the original patient tumor (PT) up to seven generations (P7) in immunocompromised mice and did not find any distinct histological differences across multiple passages (Supplementary Figure S3). All ovarian PDXs showed positive pan-cytokeratin (CK) staining, confirming the epithelial origin of the tumors. In addition, all of the primary tumors and their corresponding PDX models expressed PAX8 and WT1 markers, which is consistent with the HGSOC subtype (Figure 2C and Supplementary Figure S3). Further, we evaluated the presence of patient-derived tumor-infiltrating immune cells that were reported by others in early passages of certain PDXs [28]. All the original patients’ tumors demonstrated leukocyte infiltration, showing positive staining for human CD45 antigen. The majority of primary tumors (82%) stained positive for a CD3 antigen that is expressed on T-cell linage. In contrast, only 12% of the tumors exhibited positive staining for CD20, indicating that B cell infiltration is less prevalent in ovarian cancer (Supplementary Figure S3). The IHC analysis of PDXs revealed an almost complete loss of human immune cell infiltration. We were not able to detect CD45- or CD20-positive cells in the 17 PDX lines tested here. We observed minimal infiltration of CD3 positive cells (T-cell lineage) in three PDX models, which was only present in the first PDX passage and completely disappeared by the second passage (Supplementary Figure S3).

In summary, the 17 HGSOC PDXs retained the major histological characteristics of the original patients’ tumors, even following multiple passages in mice. As we showed here, human tumor-infiltrating immune cells are rapidly cleared from HGSOC tumorgrafts; thus, these models are not suitable for studying the components of a patient’s immune system. 

### 3.5. Analysis of the Genomic Fidelity and Stability of PDX Models

As the value of PDX models depends on their faithful representation of primary tumors, it is important to assess whether PDXs retain their genomic and phenotypic characteristics throughout propagation. Previous studies have demonstrated that PDX tumor models largely retain the genomic features of primary tumors [8,10,11]; however, some degree of genomic variation due to clonal evolution and/or genomic instability is expected following PDX propagation [29]. To evaluate the molecular landscape and the extent of genetic drift throughout propagation of PDX models of ovarian cancer, we performed a comprehensive genomic analysis of the originating patient’s tumors and their derivative PDX passages grown in mice. In addition, we interrogated genetic drift in the PDX passages affected by lymphoma contamination. The analysis included interrogation of putative functional exonic variants consisting of >250,000 single nucleotide polymorphisms (SNPs). The human genome is 99.9% identical between different individuals, with the 0.1% variability including SNPs. Among SNPs, there are rare variants occurring at very low frequency (minor allele frequency—MAF < 0.5%), more common variants (0.5% < MAF < 5%), and common SNPs (MAF > 5%) [30]. To determine the genomic fidelity and stability of the PDX models, we selected 300 common SNPs that frequently occur in the human genome (MAF of at least 40%). Analysis of SNPs with high MAF values warrants detection of significant differences in frequency of variants between unrelated tumor specimens or contaminated samples.

**Figure 3:**
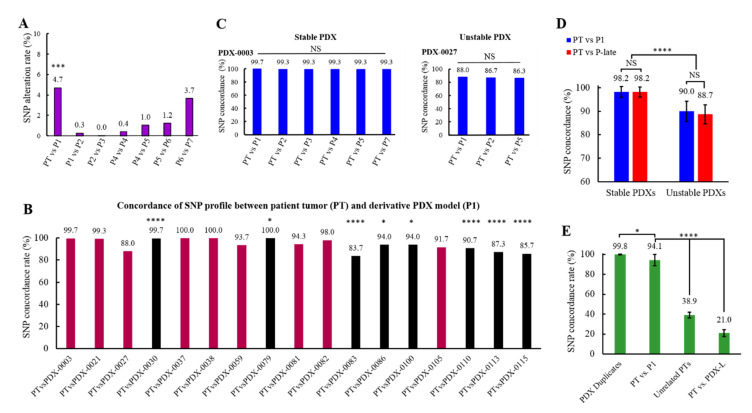
Analysis of genomic fidelity and stability of PDX models. (**A**) Graph represents SNP alteration rate in 17 PDX models across passages (pooled data are shown). Each column represents the percentage of altered SNPs between patient tumor (PT) and the consecutive PDX passage (P); (Dunnett’s test). (**B**) Graph represents a SNP concordance within each PDX by comparing originating patient’s tumor and the first PDX passage (P1). Genetically unstable PDXs are denoted by black columns (Dunnett’s test). (**C**) Comparison of SNP concordance between genetically stable and unstable PDXs presented as percentage of matching SNPs between original patient tumor and derivative PDX passages (one-way ANOVA followed by Tukey’s multiple comparisons test). (**D**) SNP concordance between patient’s tumor (PT) and initial (P1) or late (P-late) PDX passage that was evaluated in stable vs. unstable PDXs (one-way ANOVA followed by Tukey’s multiple comparisons test). (**E**) Graph shows an average SNP concordance of 17 PDXs at the time of PDX initiation (PT vs. P1), PDX duplicate samples, unrelated patients’ samples and lymphoma contaminated PDXs (one-way ANOVA followed by Tukey’s multiple comparisons test). (**A**–**E**) Data are represented as mean ± SEM. The following symbols indicate the statistical significance of data: NS = not significant, * = *p* < 0.05, *** = *p* < 0.001 and **** = *p* < 0.0001. For additional information, see Supplementary Figure S4.

Specifically, we assessed SNP alteration rate as a measure of genetic drift occurring as a result of PDX derivation and serial propagation. The analysis of 17 PDX lines revealed that genetic drift was most noticeable during PDX initiation, which is consistent with strong selection pressure associated with PDX establishment and adaptation to the mouse host [13]. We observed a significant increase in SNP alteration rate, reflected as the acquisition of 4.7% new variants in the initial PDX passage (P1) when compared with an SNP profile of the corresponding patient’s tumor (PT) (Figure 3A). Further analysis of SNP dynamics across PDX passages revealed significantly reduced rates of new variant acquisition. We detected, on average, the acquisition of 0.6% of new SNPs within six consecutive passages (P1–P6), followed by an increase in new SNP occurrence to 3.7% in passage 7 (P7). Our data indicate that passages P1 through P4 are the most stable PDX passages. We also found evidence of small continuous genomic evolution through passaging that becomes more evident in late PDX passages (P6–P7) (Figure 3A and Supplementary Figure S4).

Since we observed marked SNP alterations at the time of xenograft initiation, we then analyzed SNP concordance within each individual PDX line by comparing the originating patient’s tumor and the founding PDX passage (P1). Our data revealed significant differences in the degree of SNP variations between different PDX lines at the time of tumor initiation. We observed that 8 out 17 PDXs (referred here as unstable PDXs) showed significantly lower SNP concordance at the time of PDX initiation than the remaining (stable) PDXs (Figure 3B,C).

Next, we asked whether unstable PDXs acquire new SNPs at a higher rate when compared with stable PDXs during propagation. To test this, we compared genetic drift associated with SNP alteration dynamics in stable vs. unstable PDX lines. The results revealed that in stable PDX lines, SNP concordance as assessed between the patient’s tumor (PT) and the initial PDX passage (P1) or late passages (PT vs. P-late) remains unchanged (98.2% vs. 98.2%). These findings indicate that the genetic drift observed in stable PDXs occurs almost exclusively at the time of PDX initiation, after which the following PDX passages remain genomically stable (Figure 3D). In contrast, we observed a decrease in SNP concordance at the time of PDX establishment (PT vs. P1, 90.0%) and further reduction of SNP concordance through PDX passaging (PT vs. P-late, 88.7%) in unstable PDX lines (Figure 3D). To determine whether unstable PDX tumors harbor *BRCA* genes mutations contributing to the DNA repair deficiency and genomic instability commonly found in HGSOC subtype [31,32], we performed an analysis of the patients’ clinical data. Our data revealed that within the unstable PDX lines two models (PDX-0027 and PDX-0115) can be derived from the tumors harboring *BRCA1/2* mutations (Supplementary Table S1). Other unstable PDX lines were either negative for *BRCA1/2* mutations or not tested, which does not eliminate possibility of epigenetic silencing of *BRCA* genes or other abnormalities within the DNA repair pathway. In contrast, none of the stable PDX lines tested positive for *BRCA* mutations.

Lastly, we carried out SNP profiling experiments to identify the SNP signature, which is conserved in patient tumor specimens and their derivative PDXs and can be successfully used to distinguished unrelated PDXs samples or identify lymphoma-contaminated models. We selected 300 SNPs frequently occurring in the human genome and compared the average SNP concordance of 17 PDX models at the time of PDX initiation (PT vs. P1) with the SNP concordance of PDX duplicate samples (control), unrelated patient samples, and lymphoma-contaminated PDXs. The results revealed that the founding PDX passage (P1) matches its respective patient tumor on 94.1% of SNPs, while unrelated patients’ tumor samples or lymphoma-transformed PDXs match only on 38.9% and 21.0% of SNPs, respectively (Figure 3E and Supplementary Figure S4). These findings show that the PDX-specific SNP signature can be utilized to distinguish unrelated PDX samples and to detect PDX contamination with lymphoma with high fidelity.

Overall, our data showed that ovarian PDX lines remain largely stable throughout propagation (passages P1-P4 demonstrated the highest genomic stability). Some marginal genetic drift occurred at the time of PDX initiation, likely associated with the adaptation of human tissue to mouse hosts. We also found that several individual PDX lines are more genetically unstable than others, which may be associated with DNA repair deficiency due to *BRCA* mutations.

### 3.6. Assessment of the Cellular Composition of the HGSOC PDX Tumors

Human tumor stroma and tumor-infiltrating immune cells are replaced with mouse equivalents upon passaging of PDX tumors in vivo. The amount and the composition of stroma in PDXs derived from different tumor types is highly variable [33]. In addition, a high content of mouse cells in PDX tumors significantly impairs downstream genomic and proteomic applications. To overcome these limitations, we optimized an efficient method to evaluate the cellular content within individual PDX models by combining automated tissue dissociation with magnetic cell sorting followed by immunophenotyping of cells by flow cytometry [22,33]. In addition, this procedure allows the isolation of a pure population of human tumor cells from PDXs, enabling more effective genetic manipulation of primary tumor cells [22].

**Figure 4:**
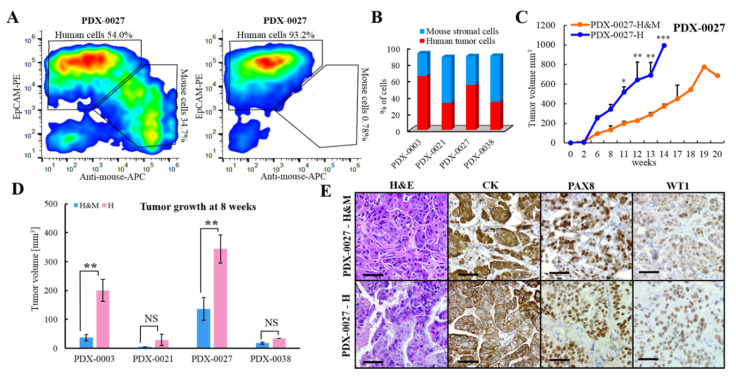
Assessment of the cellular composition of HGSOC PDXs. (**A**) Flow cytometry analysis of cell fractions before (left panel) and after (right panel) depletion of mouse cells from PDX-0027 by magnetic cell sorting. Cells were labeled with a pan-mouse antibody cocktail and a human-specific antibody against CD326. (**B**) Graph represents the percentage of human tumor cells and mouse stromal cells in individual PDX lines (passage 4). The cellular content was assessed by magnetic cell sorting of cells isolated from PDX, followed by flow cytometry analysis. (**C**) Graph shows tumor growth rate of PDX-0027 inoculated as pure population of human tumor cells (H) or human tumor and mouse stromal cells (H&M), (one way ANOVA followed by Tukey’s multiple comparisons test). (**D**) Graph shows tumor volumes at 8 weeks post-implantation of PDXs inoculated as H or H&M (unpaired t test). (**E**) Tumor sections were generated from PDXs developed from H or H&M. The sections were H&E stained and evaluated by IHC for HGSOC subtype markers. Scale bars: 50 μm. (**C**,**D**) Data are represented as mean ± SEM. The following symbols indicate the statistical significance of data: NS = not significant, * = *p* < 0.05, ** = *p* < 0.01, and *** = *p* < 0.001.

First, we estimated the stromal content of four HGSOC PDXs by performing flow cytometry analysis on cells obtained from PDX dissociation into single cells. The cell suspension was labeled with anti-CD326 (EpCAM) antibody, detecting human carcinoma cells, and/or a reagent detecting mouse cells bound to magnetic beads (Figure 4A). We observed noticeable variability in the cellular composition of different PDX lines (collected at passage 4), ranging from 32.3% to 64.9% of human tumor cells and 27.2% to 55.3% of mouse cells (Figure 4B). Since mouse cells may constitute as much as half of the cellular content of a PDX, this reinforces the need to either remove mouse cells experimentally (as described above) or to filter out mouse reads using data processing software prior to performing genomic analyses.

Here, we also performed a proof-of-concept experiment where we compared the tumor growth rate of mice implanted with PDX human tumor cells only and mice implanted with a human and mouse cell suspension from the same PDX (Figure 4C). As expected, tumors generated from a pure population of human tumor cells grew faster than those developed from a mixture of different cell types. Further experiments revealed that the PDXs with higher stromal content (PDX-0021 and PDX-0038) grew slower than the PDXs with lower stromal content (PDX-0003 and PDX-0027, S2 Data). Moreover, the PDXs with a naturally high tumor cell content (PDX-0003 and PDX-0027) reached significantly larger volumes when implanted as a pure population of tumor cells than when implanted as a mixture of tumor/stromal cells (Figure 4D). In contrast, PDXs with high stromal content (PDX-0021 and PDX-0038) grew only slightly faster when implanted as pure tumor cell population than a mixture of tumor/stromal cells (without reaching statistical significance, Figure 4D). The IHC analysis confirmed that there were no changes in tumor architecture, histology or the expression of HGSOC markers between PDXs generated from human tumor cells and the mixture of tumor/stromal cells (Figure 4E). Taking together, depletion of mouse stromal cells from slow-growing PDXs only marginally enhanced PDX tumor growth rate. These findings indicate that PDX growth dynamics depend on the intrinsic cancer cell growth rate of each individual PDX model. In addition, we observed that the fast-growing PDXs tended to have a higher percentage of human tumor cells relative to mouse stromal cells. We recognize that the presented data rely only on four PDX models, which highlights the need for caution when interpreting these findings. Nevertheless, we have shown here that the cellular content of PDX tumors can be precisely quantified, and the pure population of human tumor cells can be successfully isolated from PDXs and used for further studies. 

### 3.7. Assessment of the Correlation in Chemotherapy Response between Patients and Their Corresponding PDXs

**Figure 5:**
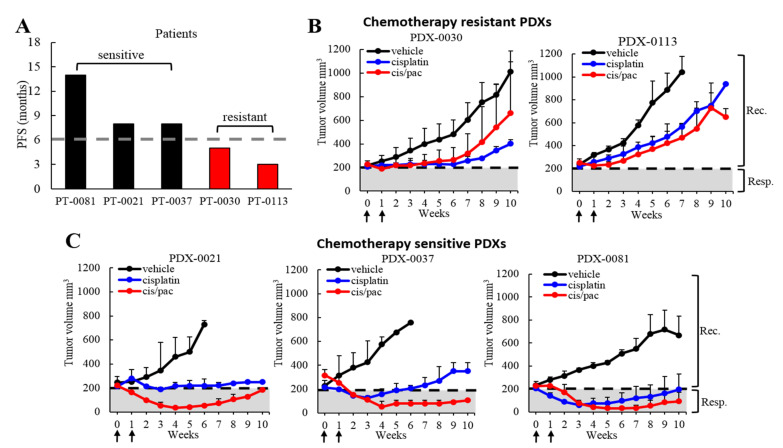
Assessment of the correlation in chemotherapy response between patients and their corresponding PDXs. (**A**) Clinical responses to platinum-based chemotherapy in patients determined by their progression-free survival (PFS). Platinum-sensitive ovarian cancer was categorized as a clinical response lasting at least six months following platinum therapy (PFS ≥ 6 months), while a platinum-resistant tumor was categorized as relapse within six months (PFS < 6 months). (**B**) Graphs show chemoresistant PDX growth rates and response to treatment. Mice received two doses of cisplatin or cisplatin+paclitaxel (arrows) or vehicle control. Rec. = recurrence, Resp. = response. (**C**) Graphs show chemosensitive PDX growth rates and response to treatment.

To determine whether our ovarian PDX lines exhibit chemotherapy response similar to that observed in their corresponding patients, five representative PDX models underwent cisplatin and/or paclitaxel treatment in vivo. Clinical responses to platinum-based chemotherapy in patients were categorized as sensitive or resistant based on their progression-free survival (PFS) (Figure 5A and Supplementary Table S1) [34].

We subcutaneously implanted five different PDX models into NOD/scid mice. We categorized our models as chemotherapy sensitive: PDX-0021, PDX-0037, PDX-0081, and chemotherapy resistant: PDX-0030 and PDX-0113, based on patients’ platinum-response data (Figure 5A). When tumors reached 200 mm^3^ volume, the animals received two cycles of cisplatin (3 mg/kg once a week) and/or paclitaxel (10 mg/kg once a week). Mice were monitored for chemotherapy response during 2 weeks of treatment and 8 weeks of follow up period (Figure 5B,C). The two chemotherapy-resistant models showed an initial tumor growth attenuation at the time of treatment followed by prompt tumor relapse at week 6 (PDX-0030) or week 2 (PDX-0113) (Figure 5B). These findings are in agreement with the respective patients’ treatment response, where PT-0030 and PT-0113 patients relapsed 5 and 3 months following chemotherapy, respectively (Figure 5A). In contrast, the three chemotherapy-sensitive PDXs demonstrated tumor regression in response to therapy that was maintained throughout the treatment and the follow up period (with the expectation of cisplatin-treated PDX-0037 that relapsed at week 7, Figure 5C). In addition, tumor-to-control (T/C) ratio calculation confirmed that PDX-0021, PDX-0037 and PDX-0081 are responsive to cisplatin and cisplatin/paclitaxel treatment, while the PDX-0030 and PDX-0113 models are nonresponsive to these therapies (Supplementary Figure S5) [35]. Collectively, our findings revealed that all five PDX models showed high concordance with the clinical responses of their corresponding patients.

### 3.8. Luciferization of PDX Models for Non-Invasive In Vivo Imaging

**Figure 6:**
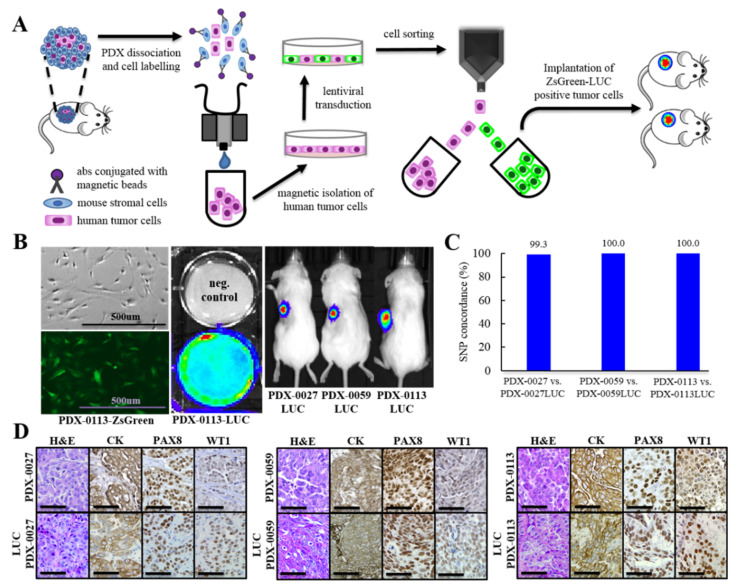
Luciferization of PDX models for non-invasive in vivo imaging. (**A**) Schematic representing a process of PDX luciferization. (**B**) Pictures show a successful transduction of primary tumor cells isolated from PDX with bicistronic lentiviral vector expressing ZsGreen (fluorescence microscopy, left panel), and expressing LUC (bioluminescence imaging of cells, middle panel). Right panel shows bioluminescence imaging of luciferized PDXs in vivo. (**C**) Analysis of 300 SNPs that frequently occur in human genome in parental PDX lines and their luciferized counterparts. (**D**) Tumor sections of PDX models with and without LUC expression were H&E stained and evaluated by IHC for HGSOC subtype markers. Scale bars: 50 μm.

The orthotopic route for tumor implantation provides the most optimal microenvironment for ovarian tumors to develop and metastasize [36]. However, orthotopically implanted ovarian cancer (e.g., into the fallopian tubes, where the HGSOC subtype originates) is difficult to precisely monitor for tumor growth and drug response [37]. Here, we aimed to develop PDX models that can be visualized by bioluminescence imaging (BLI), which is a sensitive, semi-quantitative technology for in vivo monitoring of biological events in live animals. BLI requires the genetic manipulation of tumor cells to express bioluminescent reporters such as luciferase (LUC) for in vivo visualization [23,38,39]. Primary cancer cells are difficult to establish and genetically manipulate in culture, in contrast to commercially available cancer cell lines. In addition, ~50% of PDX tumor tissue consists of infiltrating mouse stromal cells that generate an additional barrier to efficiently transducing primary tumor cells (Figure 4A,B) [22]. To express a luciferase gene in human tumor cells, we first depleted mouse stromal cells from three individual PDX lines by magnetic cell sorting [22,33]. Next, purified human tumor cells were cultured for a short term (<7 days) followed by the transduction of cells with a bicistronic lentiviral vector expressing two reporter genes, LUC and ZsGreen (Figure 6A). Five days after lentiviral transduction, the successfully transduced cells were sterile sorted for ZsGreen expression and immediately implanted into mice to generate luciferized PDXs (Figure 6B).

We expanded our luciferized PDX lines for two passages, followed by histological and molecular validation. Our data revealed that the PDX luciferization process does not affect the histology or the expression of HGSOC markers such as CK, PAX8 and WT1, which were faithfully preserved in the luciferized models (Figure 6C,D). The SNP distribution analysis revealed that luciferized xenografts are genetically stable, as reflected in high SNP concordance between pairs of parental and luciferized PDXs. We detected 100% SNP concordance between PDX-0059 and PDX-0113 vs. their corresponding luciferized models. The PDX-0027-LUC line showed a marginal decrease in SNP concordance to 99.3% when compared to its parental PDX-0027 *BRCA2* mutant line (Figure 6C). Further, we validated the luciferized PDX models in vivo to confirm their luciferized expression (Figure 6B). In summary, our newly developed luciferized PDX xenografts allow reproducible in vivo assessment of orthotopic or intraperitoneal tumor burden via bioluminescence imaging.

## 4. Discussion

PDX models are becoming the preferred pre-clinical tool in both industry and academic settings in an attempt to improve the drug development process [9,40]. Here, we present a collection of serially transplantable, extensively characterized PDX models representing a defined HGSOC subtype.

We performed successful engraftment of 33 primary HGSOC tumors into immunodeficient mice, reaching a tumor take rate of 77%, which is in an agreement with other studies reporting 74–83% engraftment rates of the HGSOC subtype [7,41]. In contrary, Liu et. al. [9] and Ricci et. al. [42] reported significantly lower epithelial ovarian cancer engraftment rates of 31% and 25%, respectively. Liu and colleagues used a different protocol than our group, utilizing tumor cells isolated from ascites rather than solid tumor fragments, while Ricci et al. developed ovarian PDXs in athymic nude mice that are only partially immunocompromised, which could explain the less efficient engraftment of human tissues [43]. In general, engraftment rates are higher in more immunodeficient mouse strains. In the literature, NSG or NRG mice are preferred over less immunocompromised strains such as athymic nude or NOD/scid mice [44,45,46]. Our study showed that there is no difference in tumor take rates between NSG or NRG mouse strains, and slightly less in immunocompromised NOD/scid mice (Fisher’s exact test, *p* = 0.4809), which is in agreement with findings reported in other studies [8]. However, we recognize the limitations of statistical analysis in assessing the factors affecting tumor engraftment rate in our study due to the small sample size, and we suggest interpreting these data with caution.

Biobanking of patient tumors and PDX tissues is essential to the development and feasibility of PDX models. One of the early studies described a method for PDX tissue cryopreservation using a medium with low FBS content (30% FBS, 10% DMSO, 60% and RPMI), which was associated with low tumor take rates [47]. Further improvement in cryopreservation techniques was achieved by increasing the FBS content in the freezing medium to 90% (90% FBS, 10% DMSO) [48,49]. In this study, we adopted a protocol which uses a high percentage of FBS in freezing medium (95% FBS, 5% DMSO) [17]. In addition, we followed rigorous protocols to complete the overall procedure of patient tumor collection and processing within 2 h. We showed here that our efficient protocols allow for successful development of PDX models from fresh as well as from previously cryopreserved tumors, which is crucial in circumstances where the patient’s tissue cannot be immediately implanted into mice following harvesting. There were no significant differences in the engraftment rates of cryopreserved vs. fresh primary tumors (65% vs. 80% shown in Table 1) in our study. Our data, together with previous studies, reinforce the importance of using FBS-rich freezing medium and reducing the ex vivo processing time of primary tumor specimens in order to reduce ischemia, which negatively affects tissue viability and engraftment success [17,48,50,51,52].

In this study, we found that the only factor affecting successful tumor engraftment rate is associated with intrinsic features of the primary tumor reflecting its aggressiveness. We showed that ovarian tumors from patients who relapsed within 12 months post-treatment engrafted significantly better than tumors harvested from patients who were cancer-free for more than 12 months following chemotherapy (Table 1). These findings are in agreement with other studies. For instance, Pergolini et al. reported that patients with pancreatic cancer that successfully engrafted had significantly shorter relapse-free survival compared to patients harboring tumors that failed to engraft [53]. In a different study, Jung et. al. demonstrated that the successful engraftment of primary lung tumors significantly correlated with shorter PFS and OS of donor patients [54]. It has also been shown that the advanced tumor stage plays a vital role in successful primary tumor engraftment. Chen Y et. al. reported that stage II and III non-small cell lung cancers engraft markedly better than stage I tumors. Similarly, Oh et al. showed that colorectal tumor take rates increased significantly with more advanced tumor stage [55]. In our study, due to the nature of HGSOC tumors, which are diagnosed at a late tumor stage (stage III and IV in most cases), we were not able to assess and compare the engraftment rates of early vs. late tumor stages. Nevertheless, our findings are in agreement with previous studies showing that there is a strong correlation between successful tumor engraftment rate and advanced tumor burden [56].

Contamination of PDX lines with lymphoproliferative lesions is a widely recognized problem in a process of PDX development. Immunodeficient mouse strains (including NOD/scid, NSG, NRG and NOG mice) are vulnerable to formation of lymphomas following human tissue xenografting [57,58,59,60]. The majority of studies reported development of Epstein–Barr virus (EBV)-associated lymphomas as a result of insufficient immune surveillance in a recipient mouse [58,60]. In our study, we identified several PDX-bearing mice whose tumors underwent lymphoma transformation associated with expansion of mouse pre-T cells. Our findings are consistent with previous reports showing that NOD/scid mice have high incidence of thymic lymphomas arising from immature pre-T thymocytes [26,27]. In contrast to others, we did not detect EBV-associated lymphomas in our study. This discrepancy could be explained by previous findings showing that high inflammation levels associated with infiltration of human immune cells in primary tumors significantly contribute to EBV-associated lymphomagenesis in the derivative PDX lines. PDXs generated from tumors with higher baseline inflammation (e.g., gastric cancers) or tumors exposed to chemotherapy-inducing immune cell infiltration are significantly more susceptible to EBV-associated lymphomas [25]. Our ovarian PDXs were developed from chemotherapy-naïve tumors showing marginal infiltration of human immune cells (minimal CD45 staining, Supplementary Figure S3); thus, we observed a particularly low incidence of EBV-associated lymphomas. Nevertheless, our routine protocols are designed to test PDX models for contamination with both mouse- and human-derived lymphoproliferations.

Multiple studies have demonstrated that PDX models recapitulate key features of the original tumors with high fidelity [7,8,9,10,11]. However, there are concerns that PDXs may evolve and change on the molecular level throughout propagation [12,13]. To address these challenges, we performed SNP genotyping of primary tumor samples and their derivative PDXs to evaluate the genomic fidelity and stability of those models. The PDX lines investigated here largely retain the genomic features of primary tumors, as has been shown by others [8,10,11,61,62]; however, we observed some degree of genomic variation and instability. The genomic drift was most noticeable during PDX initiation, showing the highest SNP alteration rate (4.7%) between the patient’s tumor (PT) and the first PDX passage (P1). Further PDX passages were significantly more stable genetically (SNP alteration rate of P1 through P4: 0.0–0.4%). Similar to our work, other studies have developed SNP-based PDX authentication and/or genomic stability validation systems showing that SNP profiles are conserved and stable in individual PDX lines [10,62,63]. Ben-David et. al. observed that copy number alterations (CNAs) mostly occur during early PDX passages, and the rate of model-acquired CNAs decreases throughout propagation [13]. These findings reflect the observation that changing the tumor microenvironment (from human to mouse) generates selective pressure which is strongest at the time of PDX initiation, allowing the fittest cancer cells to adapt to the mouse host [13].

Further, in our work, the comparison of SNP alteration rates between different PDX models identified several unstable PDX lines characterized by more pronounced genetic drift, which could be associated with *BRCA* mutations. *BRCA* mutations contribute to DNA repair pathway deficiency and genomic instability, which is prevalent in HGSOC patients [31,32]. However, as we had only two PDX models harboring *BRCA* mutations, our findings should be interpreted with caution. We hope that our findings will encourage further studies to investigate the impact of *BRCA* mutations on genomic stability and genetic drift in PDX models.

In this work, we also developed a 300-SNP signature that can be successfully utilized to distinguish unrelated PDX samples, detect PDX contamination with lymphoma with high fidelity, and track subtle molecular changes across PDXs during passaging. Similar findings have been reported by El-Hoss et. al., who demonstrated that an SNP-based PDX authentication system can serve as an effective tool to track patient tissue and identify mixed samples [62]. With the rapidly growing utilization of PDX models in cancer research, implementation of a practical, accurate, and cost-effective genotyping system for PDX authentication would improve the integrity of data generated from PDX studies.

Further, we performed an extensive analysis of histology, molecular landscape and growth kinetics across multiple PDX passages. While developing PDX lines, we observed the presence of human immune cells within primary ovarian tumors which were completely eliminated in almost all PDX lines by the first passage in the mouse host. Accordingly, the first and the following PDX passages (P1-P7) show loss of human immune cell infiltration while preserving consistent stromal content and architecture. These findings are in agreement with other reports showing that human tumor stroma is replaced with mouse equivalents and human tumor-infiltrating immune cells are rapidly cleared from PDXs upon propagation [64,65]. In contrary, Pu et. al. demonstrated co-existence of patient-derived immune cells in early passages (P1–P2) of lung cancer PDXs, suggesting that early PDX passages could be used for studying the functionality of the immune system [28]. As discussed earlier, some tumor types are characterized by higher levels of human immune cell infiltration, which on the one hand allows the study of the human immune microenvironment in early PDX passages; however, on the other hand, it increases susceptibility to EBV-associated lymphoproliferation, limiting utilization of such PDXs.

Some studies have reported a reduction of stromal content in the later passages of PDX models derived from head and neck tumors and cervical cancer, which correlates with increased PDX aggressiveness [66,67]. We also found that PDXs with lower stromal content grew faster than PDXs with higher stromal content. In addition, we observed marked variability in stromal composition between individual PDX lines, which was maintained unchanged throughout multiple passages. It is possible that faster-growing PDX models consist of more aggressive tumor cells that proliferate at higher rates, generating tumor mass with less stroma. It is also likely that the growth of PDX results from a balance of the aggressiveness of tumor cells and the repressive nature of the murine stromal cells. In our study, we also noticed differences in PDX tumor growth kinetics between different PDX passages, mostly attributed to the significantly slower tumor growth rate of the initial human-to-mouse passage compared to later PDX passages. Similar observations were also reported by others, showing that the first PDX passage derived directly from human tumor required a longer latency time to develop and grow when compared with subsequent PDX passages [68,69].

Chemotherapy resistance is a major challenge in the successful treatment of ovarian cancer patients [34,70,71]. Another challenge associated with overcoming chemotherapy resistance is the scarcity of in vivo models which faithfully recapitulate human tumor biology and drug response to enhance the translation of new therapies from pre-clinical models to patients. Our data demonstrated that the PDX models investigated here show high concordance with the clinical responses of their corresponding patients. Our findings are in agreement with several retrospective studies which compared patient responses to anticancer therapy with those of corresponding PDXs and reported comparable treatment outcomes in various tumor types [6,7,10,72,73].

In summary, our HGSOC PDXs retain characteristics of the original tumors, including tumor architecture, morphology and drug response, and remain genomically stable throughout propagation. However, like any other model system, understanding the limitations of the PDX model is necessary for optimal pre-clinical application. Our studies indicate that HGSOC models are not suitable for testing immunotherapies and need to be routinely authenticated and rigorously tested for potential lymphoproliferative contaminations. 

## 5. Conclusions

Our data underline the value of PDXs representing the defined HGSOC subtype. Our findings demonstrate the potential of these models to become a powerful tool in precision medicine for ovarian cancer.

## Figures and Tables

**Table 1:** Patient characteristics and PDX engraftment rate.

PATIENT POPULATION				
Characteristics	Total	Engrafted	Failed	*p* Value
No. of patients	43 (100%)	33 (77%)	10 (23%)	
Age at collection	62.2	63.0	59.5	0.3100
Stage				
IIC	1 (2%)	0 (0%)	1 (10%)	0.2326
IIIC	33 (77%)	27 (82%)	6 (60%)	0.2056
IVA	2 (5%)	1 (3%)	1 (10%)	0.4153
IVB	7 (16%)	5 (15%)	2 (20%)	1.0000
Platinum response				
resistant	15 (37%)	11 (35%)	4 (40%)	1.0000
sensitive	26 (63%)	20 (65%)	6 (60%)	1.0000
Recurrence (months)	8.3	7.4	11.0	0.0931
<12 months	29 (81%)	22 (89%)	7 (56%)	**0.0497**
>12 months	7 (19%)	3 (11%)	4 (44%)	
Overall survival (months)	24.5	24.8	22.6	0.6908
0–24 months	17 (52%)	13 (48%)	4 (67%)	0.6562
24–36 months	11 (33%)	9 (33%)	2 (33%)	1.0000
>36 months	5 (15%)	5 (19%)	0 (0%)	0.5563
**ENGRAFTMENT METHOD**				
**Characteristic**	**Total**	**Engrafted**	**Failed**	***p* Value**
NOD/scid mice	17 (100%)	12 (71%)	5 (29%)	0.4809
NRG/NSG mice	26 (100%)	21 (81%)	5 (19%)	
Tumor preservation				
Fresh	10 (100%)	8 (80%)	2 (20%)	0.6822
Frozen/Thawed	23 (100%)	15 (65%)	8 (35%)	

Bold values denote statistical significance at the *p* < 0.05 level (Fisher’s exact test).

## Data Availability

The data presented in this study are contained within the article or Supplementary Materials. Additional data supporting the findings of this study are available from the corresponding author upon reasonable request.

## Supplementary Materials

The following are available online at https://www.mdpi.com/article/10.3390/cancers13246288/s1, Figure S1: Lymphoma transformation in PDX tumor models. Figure S2: Analysis of PDX tumor growth rates, Figure S3: IHC characterization of PDXs. Figure S4: Analysis of molecular landscape and genetic drift of individual ovarian PDX models throughout their derivation and serial propagation. Figure S5. Treatment response values (T/C) of PDX tumor models. Table S1: Clinicopathological characteristics of study patients diagnosed with high-grade serous ovarian cancer (HGSOC), Table S2: Summary of engraftment rates of PDXs.
