# Supplementary material for: Patient-Derived Xenografts of High-Grade Serous Ovarian Cancer Subtype as a Powerful Tool in Pre-Clinical Research
Source: Cancers (Basel). 2021 Dec 15;13(24):6288. doi: 10.3390/cancers13246288 (PMC8699796; doi:10.3390/cancers13246288)
Supplement: Supplementary file 1 [file cancers-13-06288-s001.zip › cancers-1483014-supplementary.pdf]

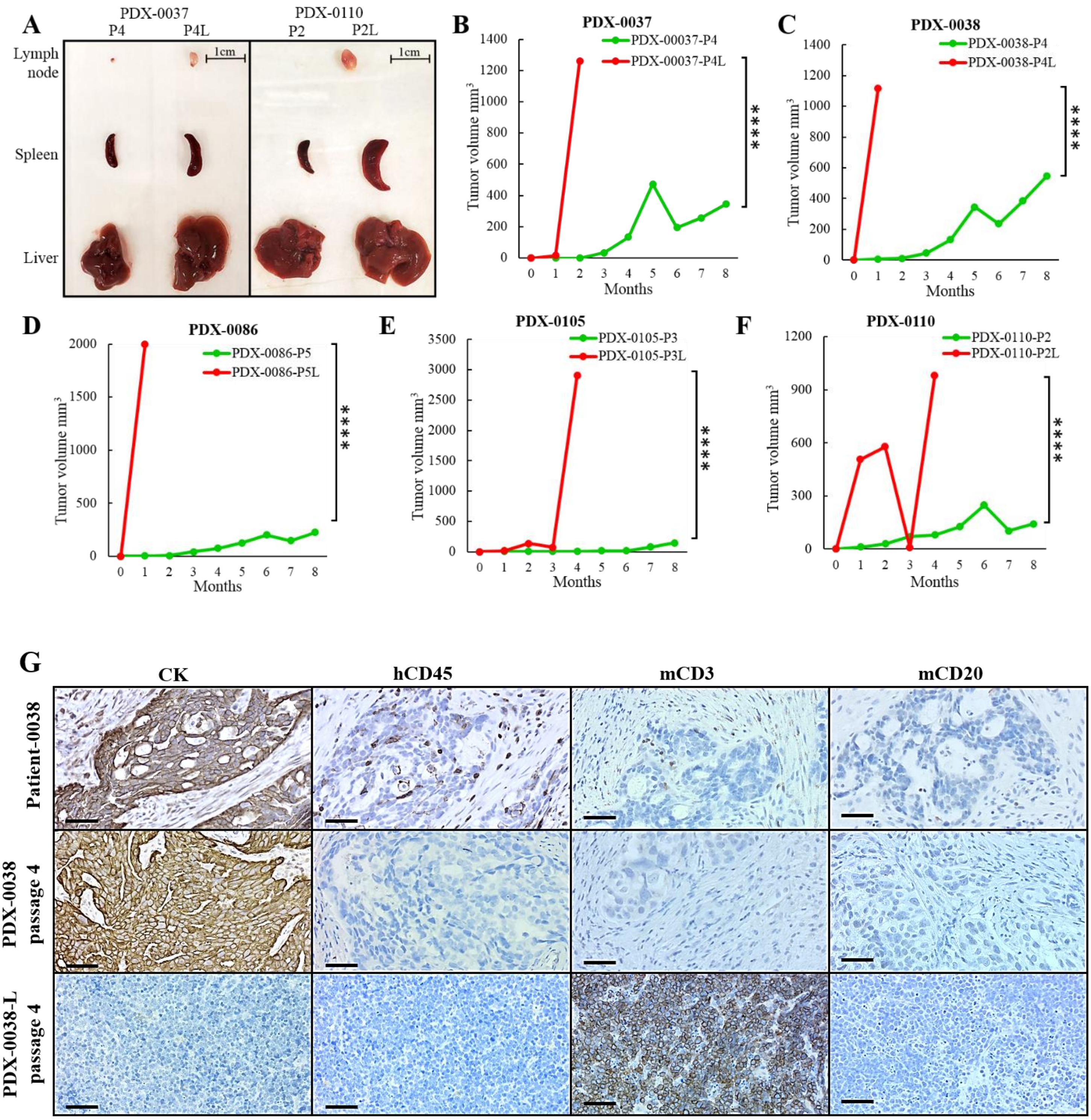
A
PDX-0037
P4
P4L
PDX-0110
P2
P2L
Lymph node
Spleen
Liver
1cm
B
PDX-0037
PDX-00037-P4
PDX-00037-P4L
Tumor volume mm3
Months
****
C
PDX-0038
PDX-0038-P4
PDX-0038-P4L
D
PDX-0086
PDX-0086-P5
PDX-0086-P5L
E
PDX-0105
PDX-0105-P3
PDX-0105-P3L
F
PDX-0110
PDX-0110-P2
PDX-0110-P2L
G
CK
hCD45
mCD3
mCD20
Patient-0038
PDX-0038 passage 4
PDX-0038-L passage 4

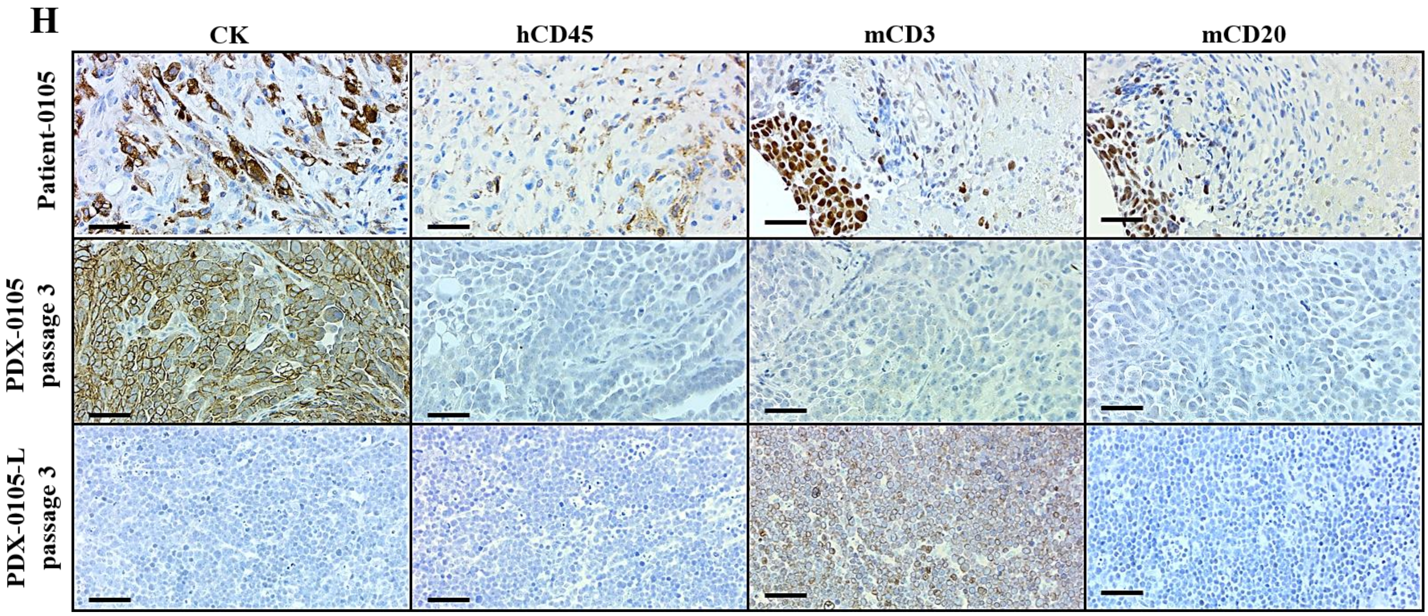

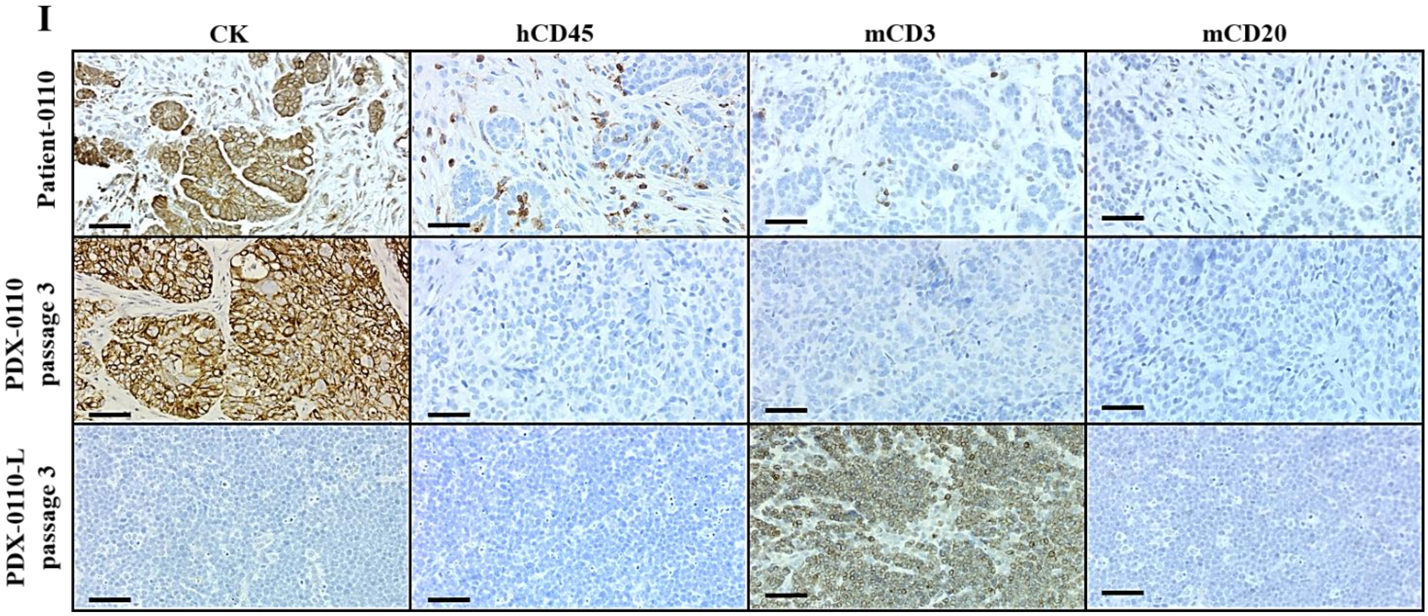

Figure S1. Lymphoma transformation in PDX tumor models. (A) Representative photographs of organs collected from lymphoma affected mouse vs. healthy control. Macroscopic analysis of lymphoma bearing mouse revealed splenomegaly, enlarged liver and lymph nodes. (B-F) Graphs represent tumor growth rate of PDXs with and without lymphoma contamination in NOD/scid mice (one way ANOVA, **** = $p<0.0001$). (G-I) Representative sections of lymphoma contaminated PDXs compared with original patient tumor and the non-affected PDX model (L = lymphoma contaminated). Tumor sections were evaluated by IHC for CK, hCD45, CD3 and CD20 expression. Scale bars represents 50 μm.

A

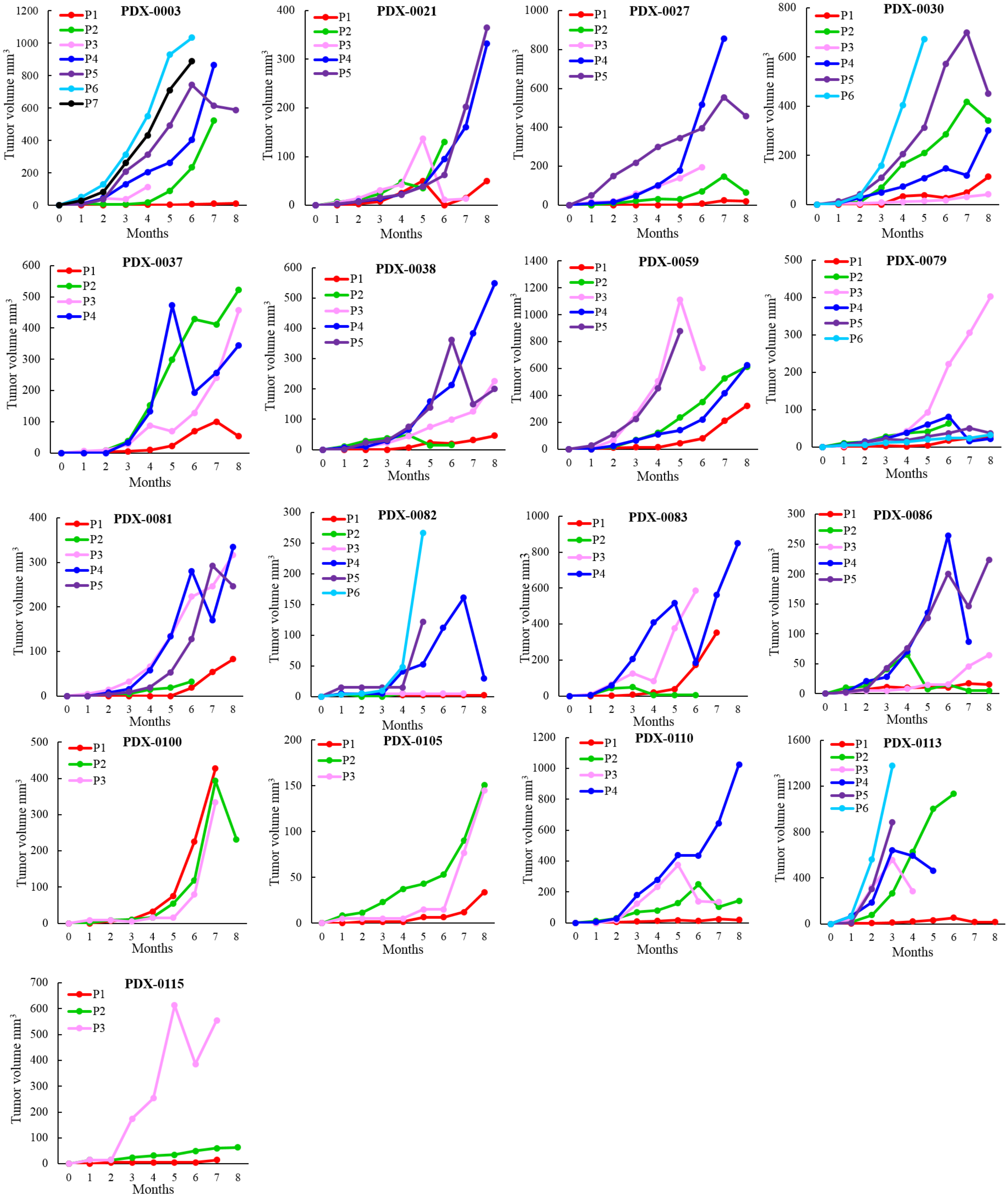
PDX-0003
P1
P2
P3
P4
P5
P6
P7
Tumor volume mm3
Months
PDX-0021
P1
P2
P3
P4
P5
Tumor volume mm3
Months
PDX-0027
P1
P2
P3
P4
P5
Tumor volume mm3
Months
PDX-0030
P1
P2
P3
P4
P5
P6
Tumor volume mm3
Months
PDX-0037
P1
P2
P3
P4
Tumor volume mm3
Months
PDX-0038
P1
P2
P3
P4
P5
Tumor volume mm3
Months
PDX-0059
P1
P2
P3
P4
P5
Tumor volume mm3
Months
PDX-0079
P1
P2
P3
P4
P5
P6
Tumor volume mm3
Months
PDX-0081
P1
P2
P3
P4
P5
Tumor volume mm3
Months
PDX-0082
P1
P2
P3
P4
P5
P6
Tumor volume mm3
Months
PDX-0083
P1
P2
P3
P4
Tumor volume mm3
Months
PDX-0086
P1
P2
P3
P4
P5
Tumor volume mm3
Months
PDX-0100
P1
P2
P3
Tumor volume mm3
Months
PDX-0105
P1
P2
P3
Tumor volume mm3
Months
PDX-0110
P1
P2
P3
P4
Tumor volume mm3
Months
PDX-0113
P1
P2
P3
P4
P5
P6
Tumor volume mm3
Months
PDX-0115
P1
P2
P3
Tumor volume mm3
Months

B

Time in months required to reach 100 mm³ tumor volume of each passage of respective PDX.

PDX-0003		PDX-0021		PDX-0027		PDX-0030		PDX-0037		PDX-0038		PDX-0059		PDX-0079		PDX-0081		PDX-0082		PDX-0083		PDX-0086		PDX-0100		PDX-0105		PDX-0110		PDX-0113		PDX-0115	
P1	8.0	P1	6.5	P1	9.5	P1	8.3	P1	7.5	P1	9.2	P1	7.7	P1	9.6	P1	7.8	P1	10.0	P1	4.0	P1	8.5	P1	6.8	P1	8.0	P1	9.8	P1	6.8	P1	8.5
P2	5.3	P2	5.0	P2	6.9	P2	3.9	P2	4.5	P2	4.0	P2	4.3	P2	8.0	P2	6.5	P2	8.0	P2	3.9	P2	5.5	P2	5.5	P2	7.5	P2	4.1	P2	2.5	P2	9.5
P3	3.0	P3	5.7	P3	4.0	P3	9.5	P3	5.4	P3	6.6	P3	2.7	P3	4.2	P3	6.0	P3	8.0	P3	2.7	P3	8.0	P3	5.5	P3	7.0	P3	3.5	P3	1.9	P3	3.6
P4	3.0	P4	6.4	P4	4.3	P4	5.8	P4	5.0	P4	4.8	P4	4.4	P4	6.8	P4	5.2	P4	6.0	P4	3.1	P4	4.7					P4	3.0	P4	1.7		
P5	2.7	P5	6.8	P5	2.8	P5	3.3			P5	4.8	P5	2.2	P5	7.8	P5	6.8	P5	5.0			P5	3.8							P5	1.7		
P6	2.1					P6	3.2							P6	8.0			P6	4.8											P6	1.3		
P7	3.3																																

C

The latency time to develop a 100 mm³ tumor

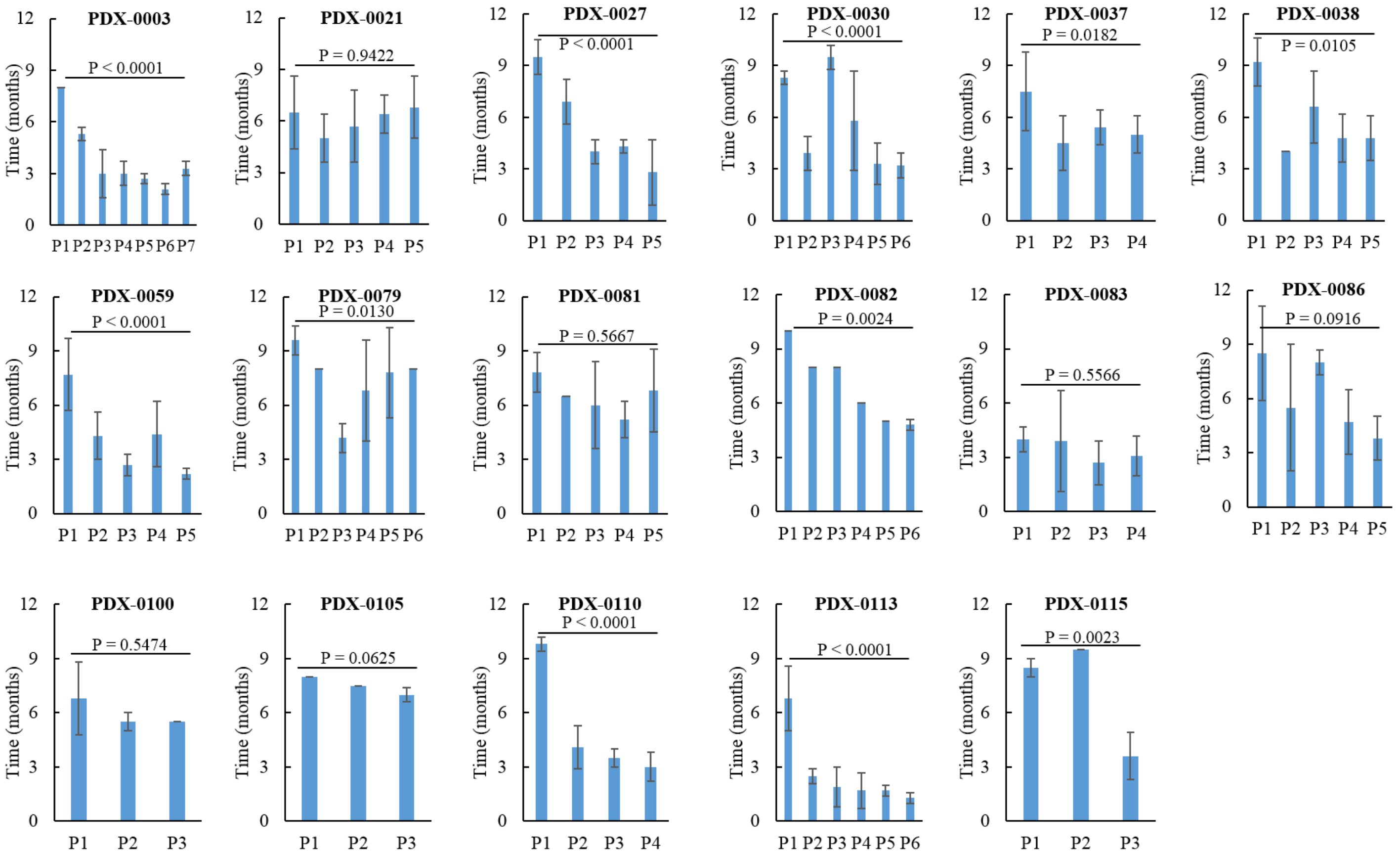

Figure S2. Analysis of PDX tumors growth rates. (A) Analysis of tumor growth rates of 17 PDX models across multiple *in vivo* passages. Graphs show growth rates of individual passages within each HGSOC PDX model. (B-C) Data presented in table (B) and graphs (C) illustrate the latency time to develop a palpable tumor (~100 mm^3 volume) from the time of initial implantation of each passage of every PDX line in this study. Data are represented as mean ± SEM (one way ANOVA).

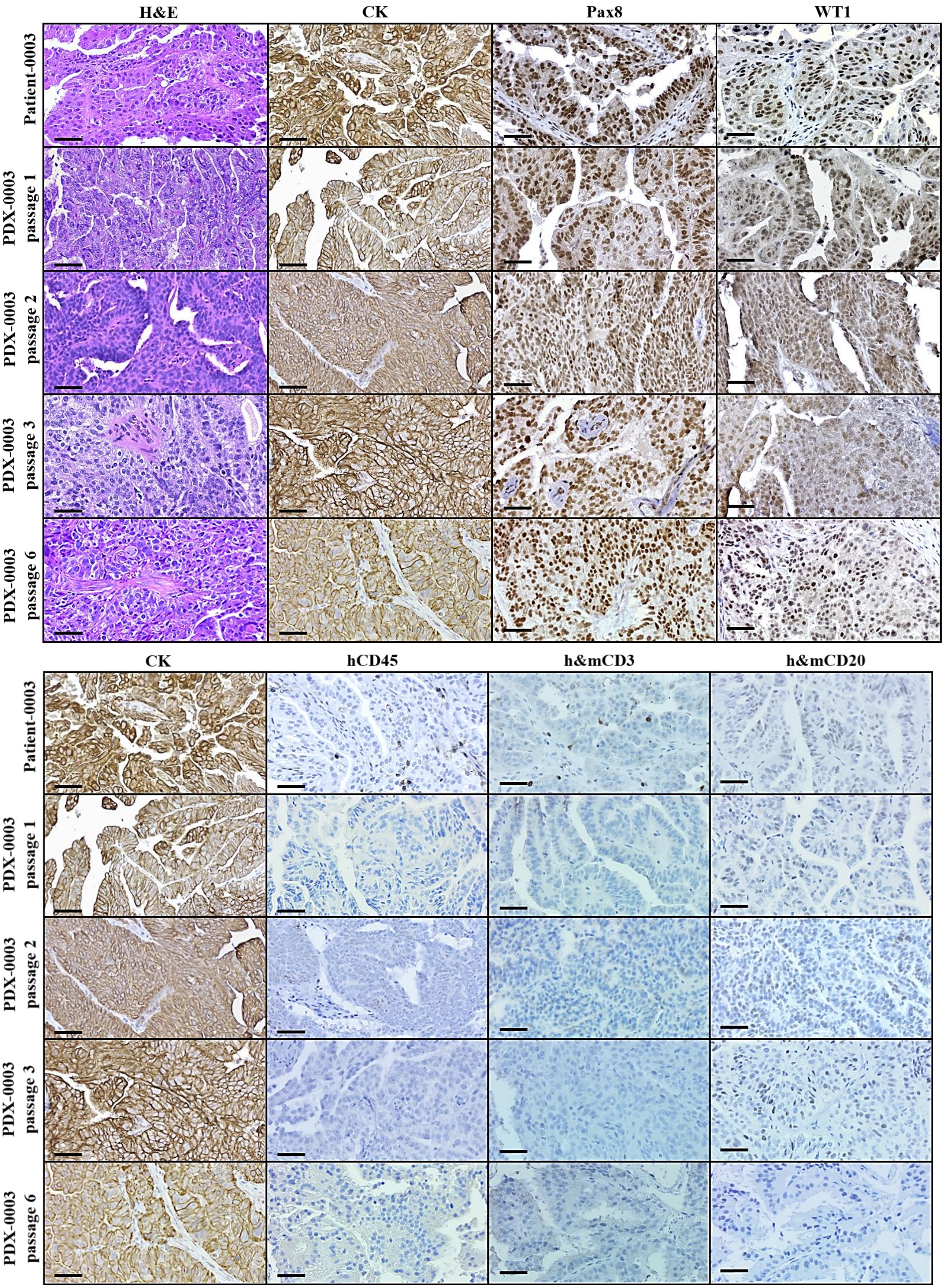
H&E
CK
Pax8
WT1
Patient-0003
PDX-0003 passage 1
PDX-0003 passage 2
PDX-0003 passage 3
PDX-0003 passage 6
CK
hCD45
h&mCD3
h&mCD20
Patient-0003
PDX-0003 passage 1
PDX-0003 passage 2
PDX-0003 passage 3
PDX-0003 passage 6

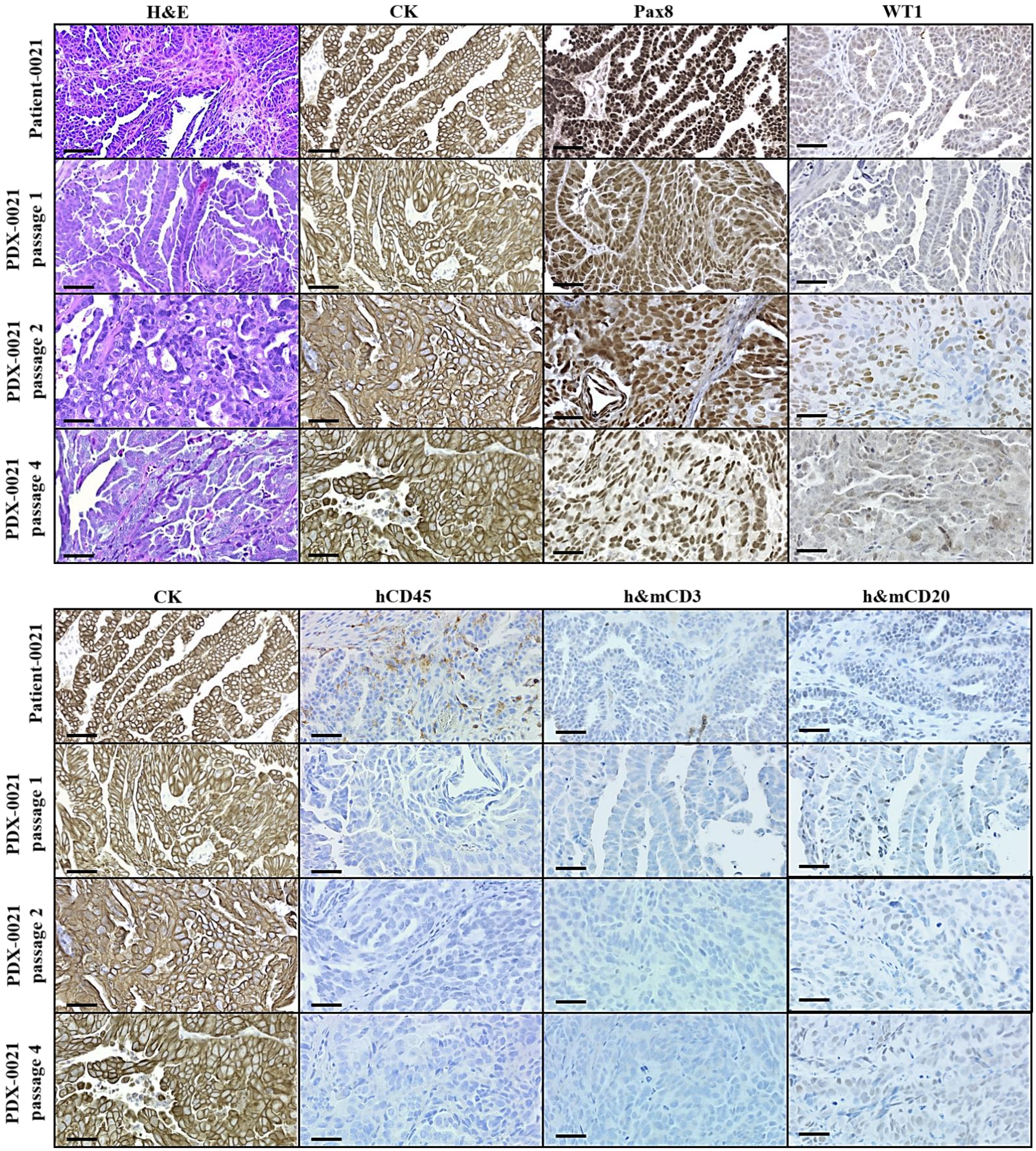
H&E
CK
Pax8
WT1
Patient-0021
PDX-0021 passage 1
PDX-0021 passage 2
PDX-0021 passage 4
CK
hCD45
h&mCD3
h&mCD20
Patient-0021
PDX-0021 passage 1
PDX-0021 passage 2
PDX-0021 passage 4

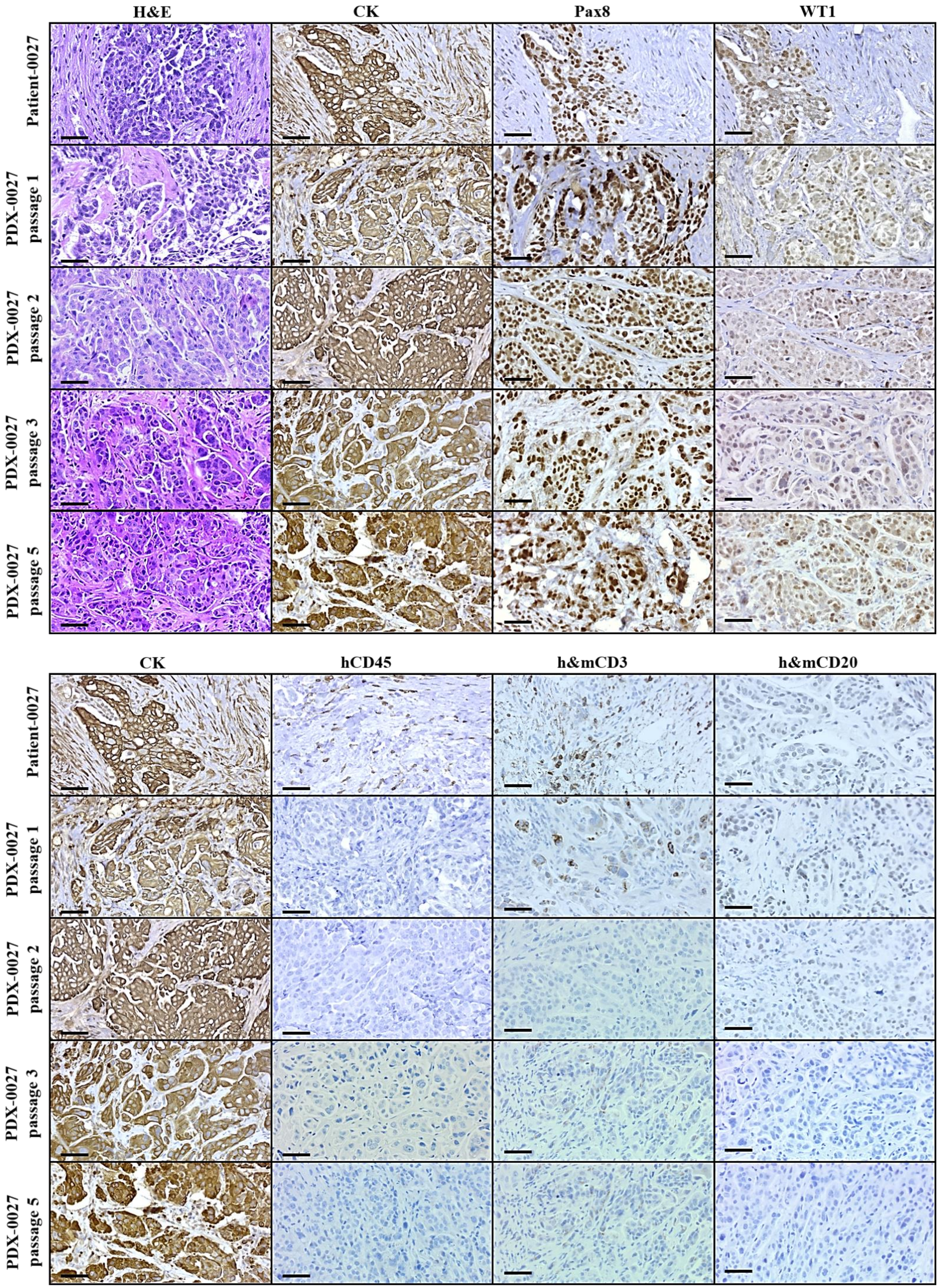
H&E
CK
Pax8
WT1
Patient-0027
PDX-0027 passage 1
PDX-0027 passage 2
PDX-0027 passage 3
PDX-0027 passage 5
CK
hCD45
h&mCD3
h&mCD20
Patient-0027
PDX-0027 passage 1
PDX-0027 passage 2
PDX-0027 passage 3
PDX-0027 passage 5

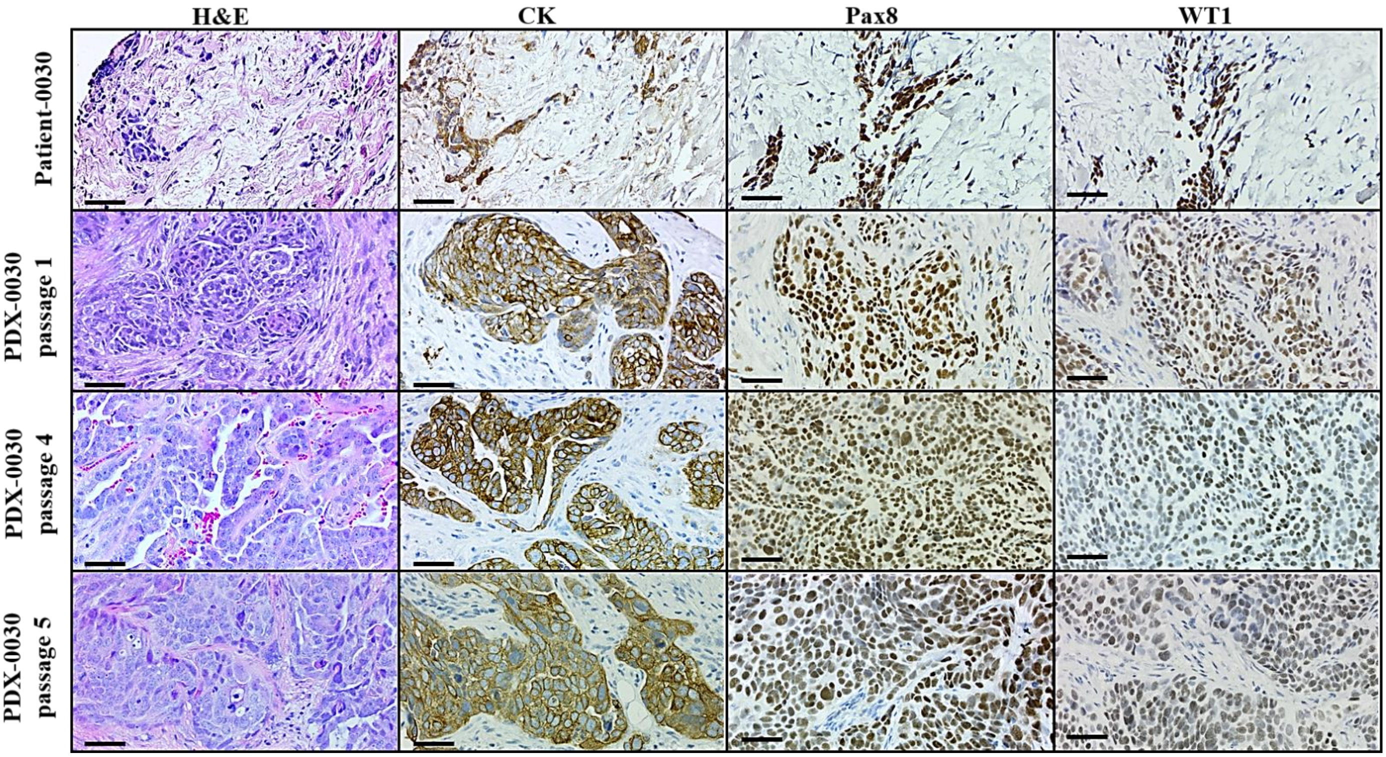
H&E
CK
Pax8
WT1
Patient-0030
PDX-0030 passage 1
PDX-0030 passage 4
PDX-0030 passage 5

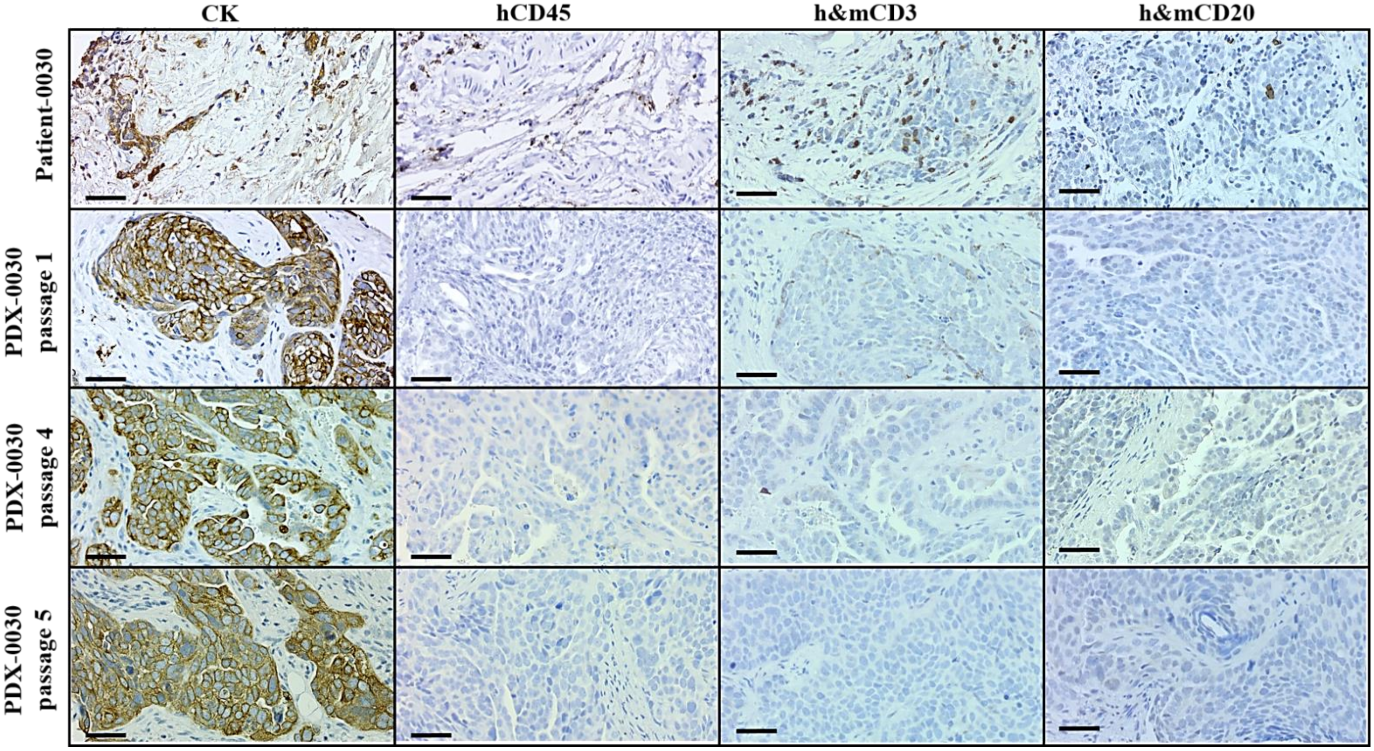
CK
hCD45
h&mCD3
h&mCD20
Patient-0030
PDX-0030 passage 1
PDX-0030 passage 4
PDX-0030 passage 5

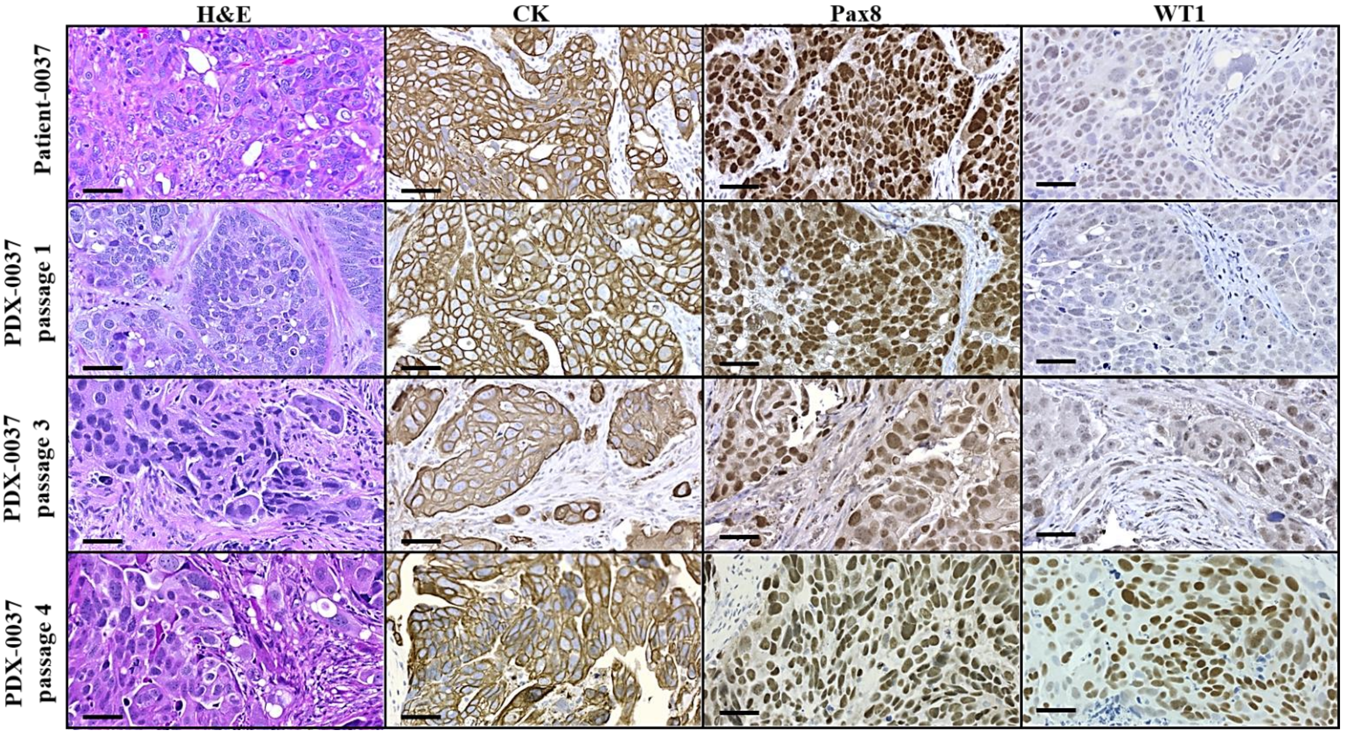
H&E
CK
Pax8
WT1
Patient-0037
PDX-0037 passage 1
PDX-0037 passage 3
PDX-0037 passage 4

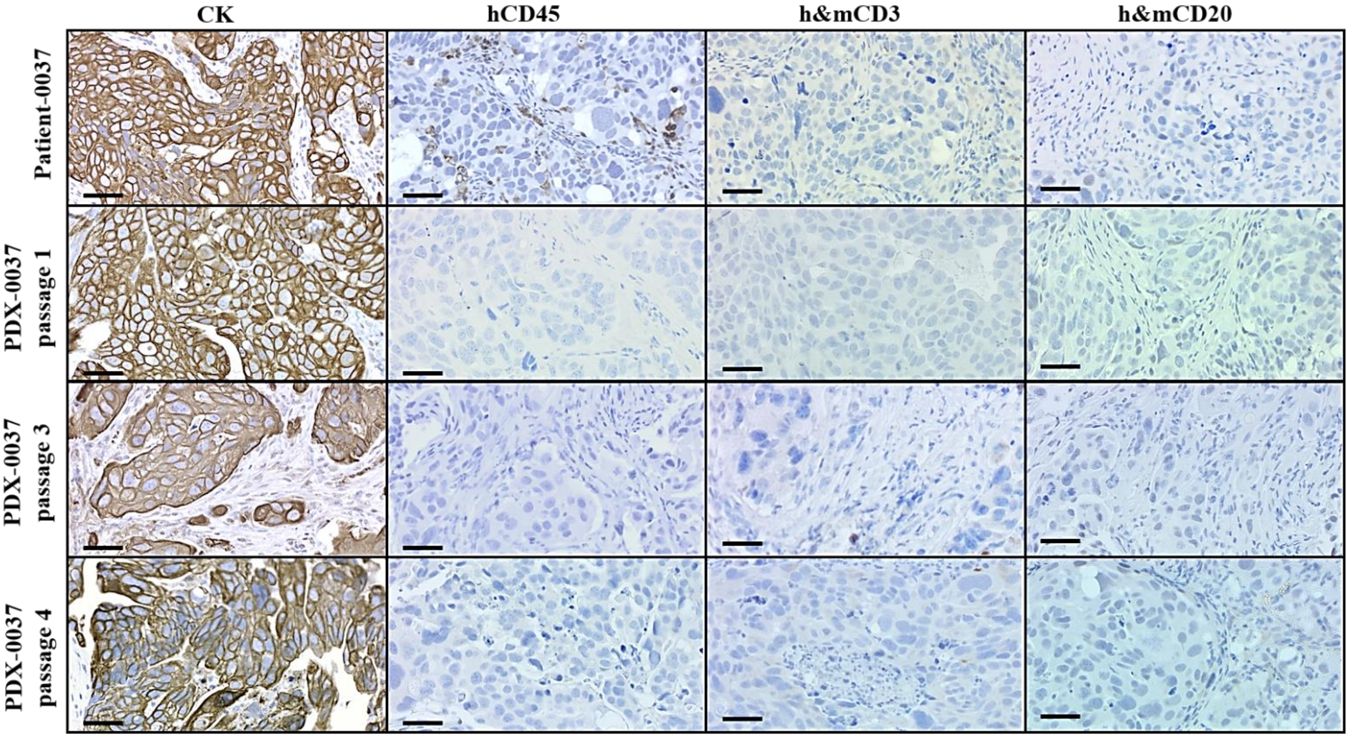
CK
hCD45
h&mCD3
h&mCD20
Patient-0037
PDX-0037 passage 1
PDX-0037 passage 3
PDX-0037 passage 4

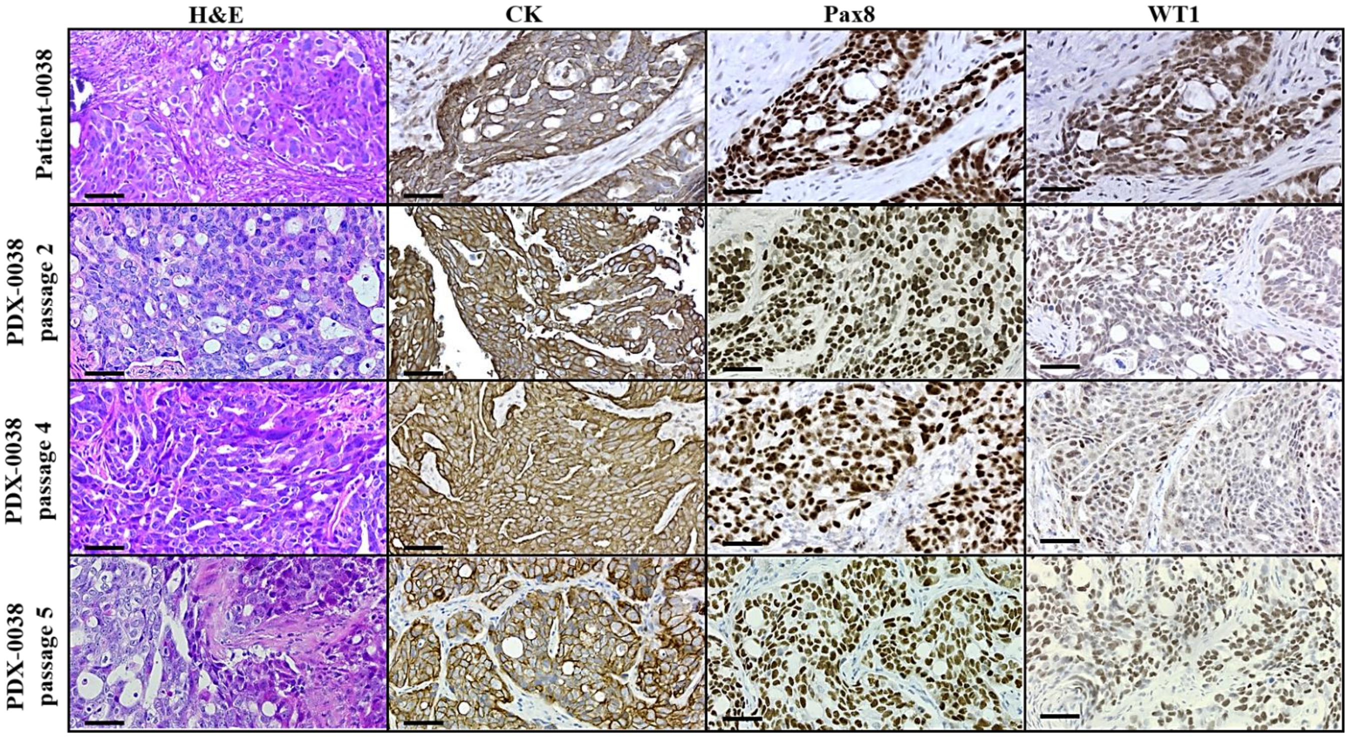
H&E
CK
Pax8
WT1
Patient-0038
PDX-0038 passage 2
PDX-0038 passage 4
PDX-0038 passage 5

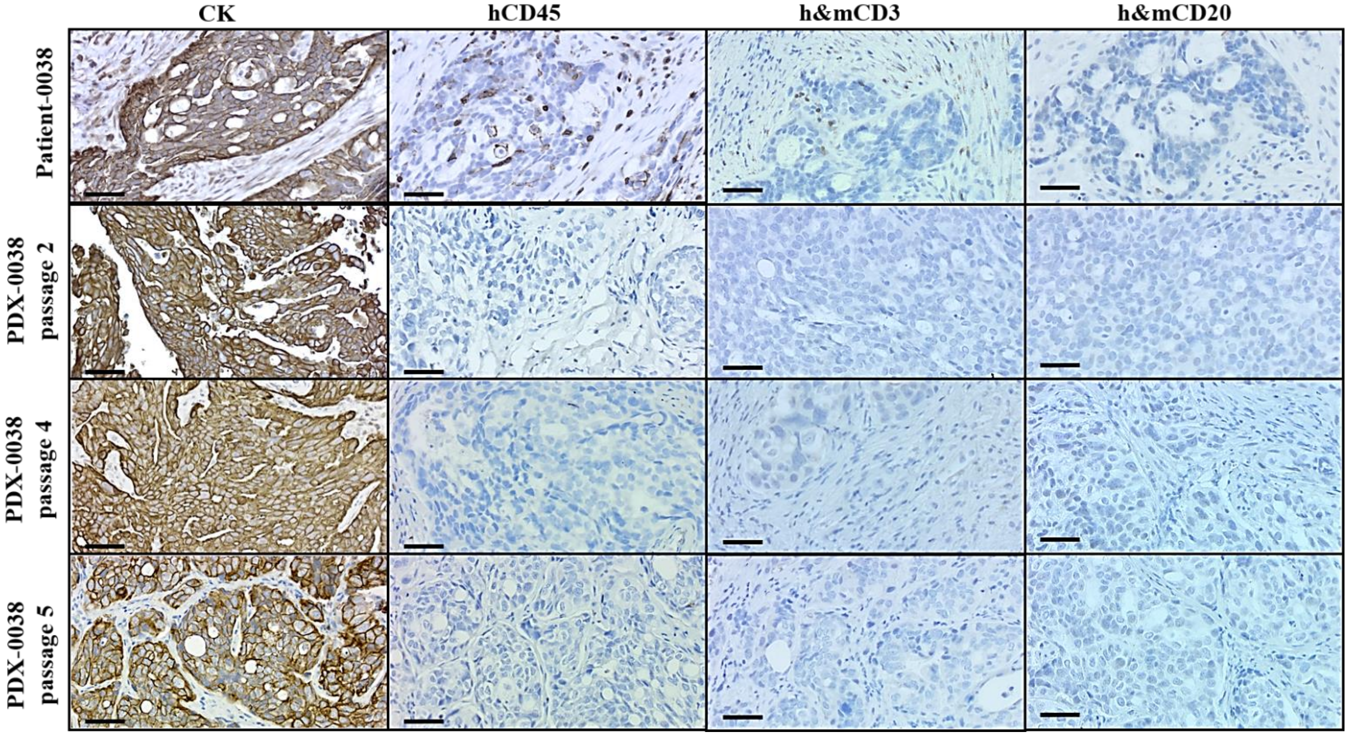
CK
hCD45
h&mCD3
h&mCD20
Patient-0038
PDX-0038 passage 2
PDX-0038 passage 4
PDX-0038 passage 5

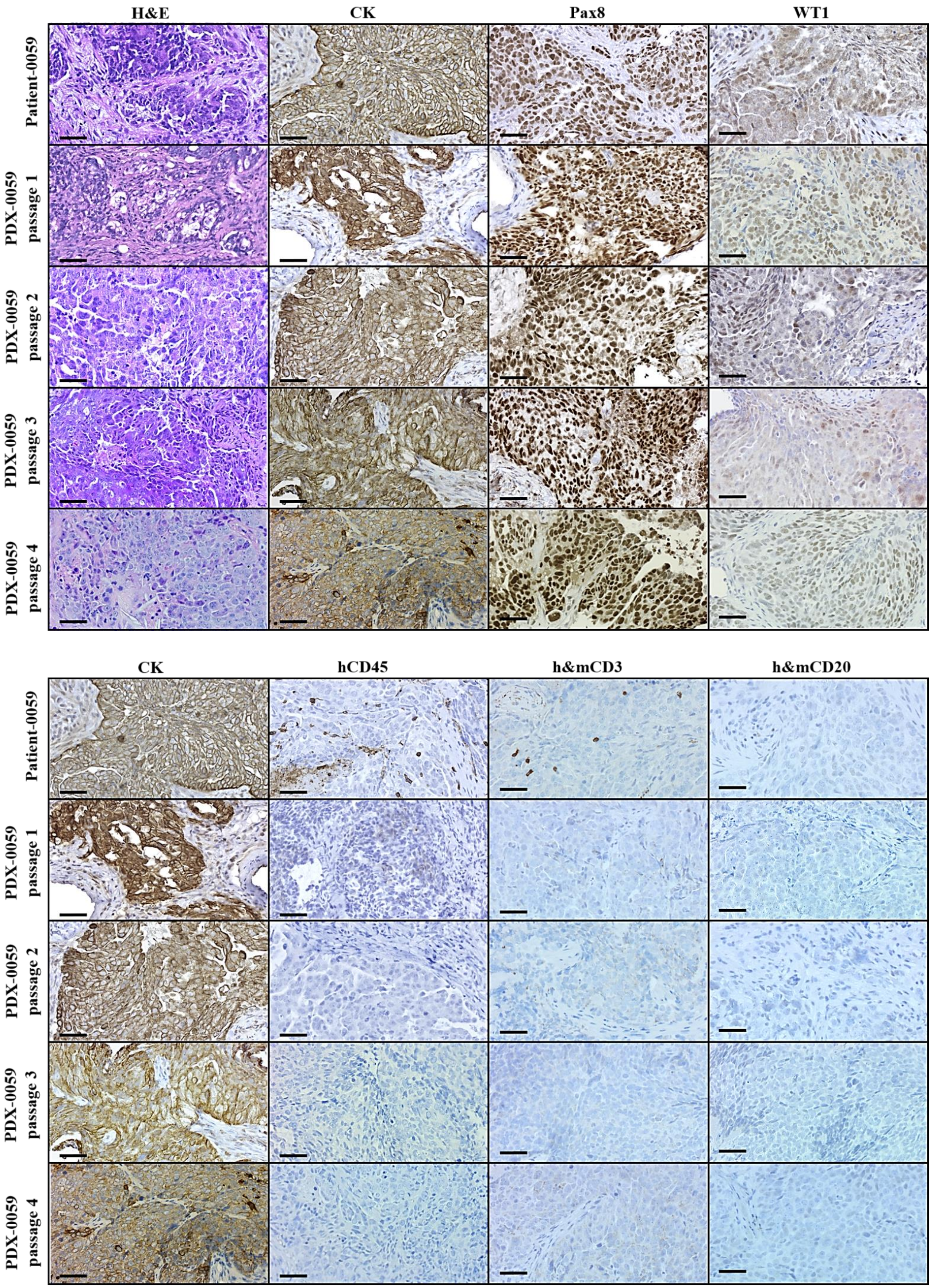

H&E
CK
Pax8
WT1
Patient-0059
PDX-0059 passage 1
PDX-0059 passage 2
PDX-0059 passage 3
PDX-0059 passage 4
CK
hCD45
h&mCD3
h&mCD20
Patient-0059
PDX-0059 passage 1
PDX-0059 passage 2
PDX-0059 passage 3
PDX-0059 passage 4

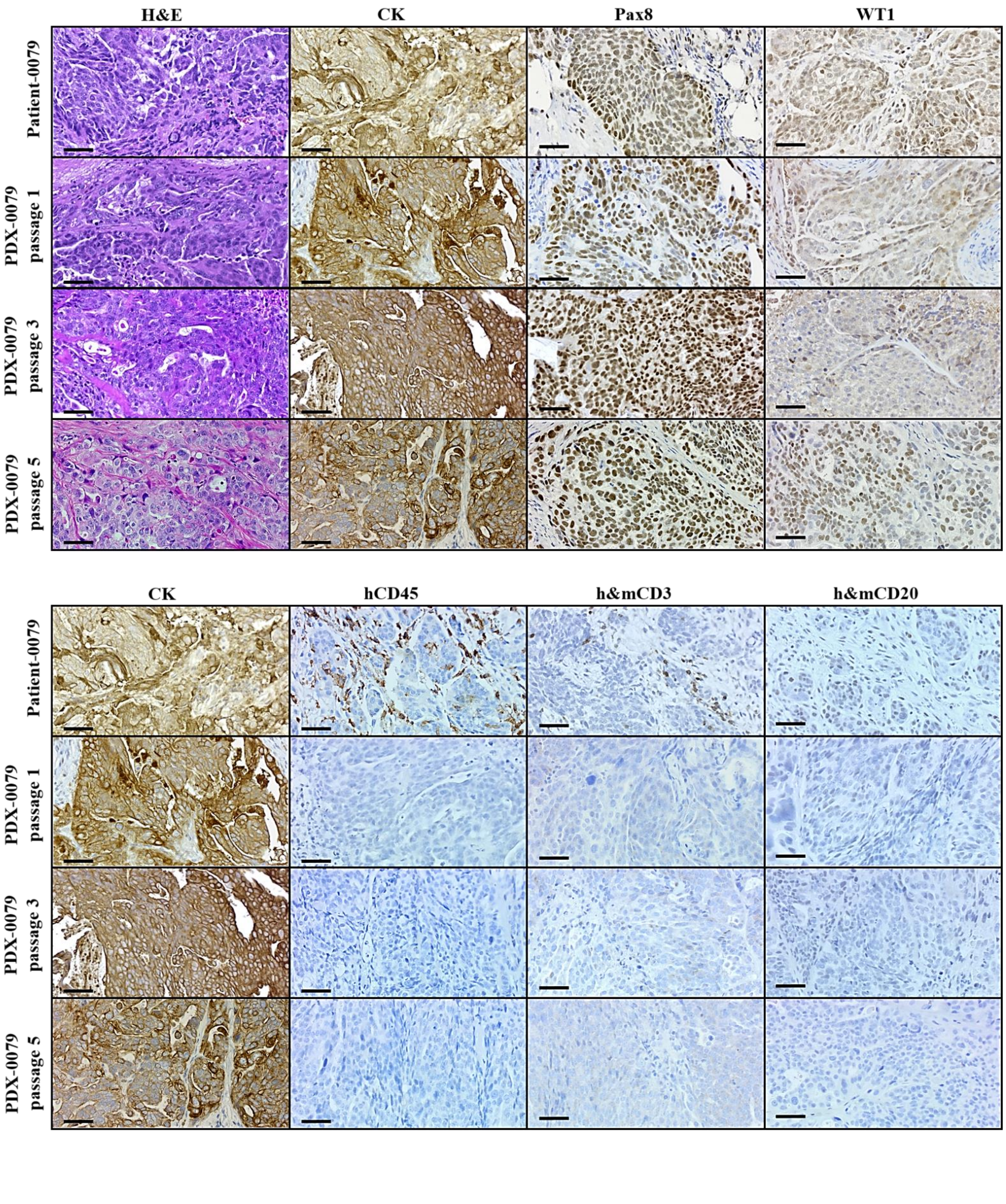
H&E
CK
Pax8
WT1
Patient-0079
PDX-0079 passage 1
PDX-0079 passage 3
PDX-0079 passage 5
CK
hCD45
h&mCD3
h&mCD20
Patient-0079
PDX-0079 passage 1
PDX-0079 passage 3
PDX-0079 passage 5

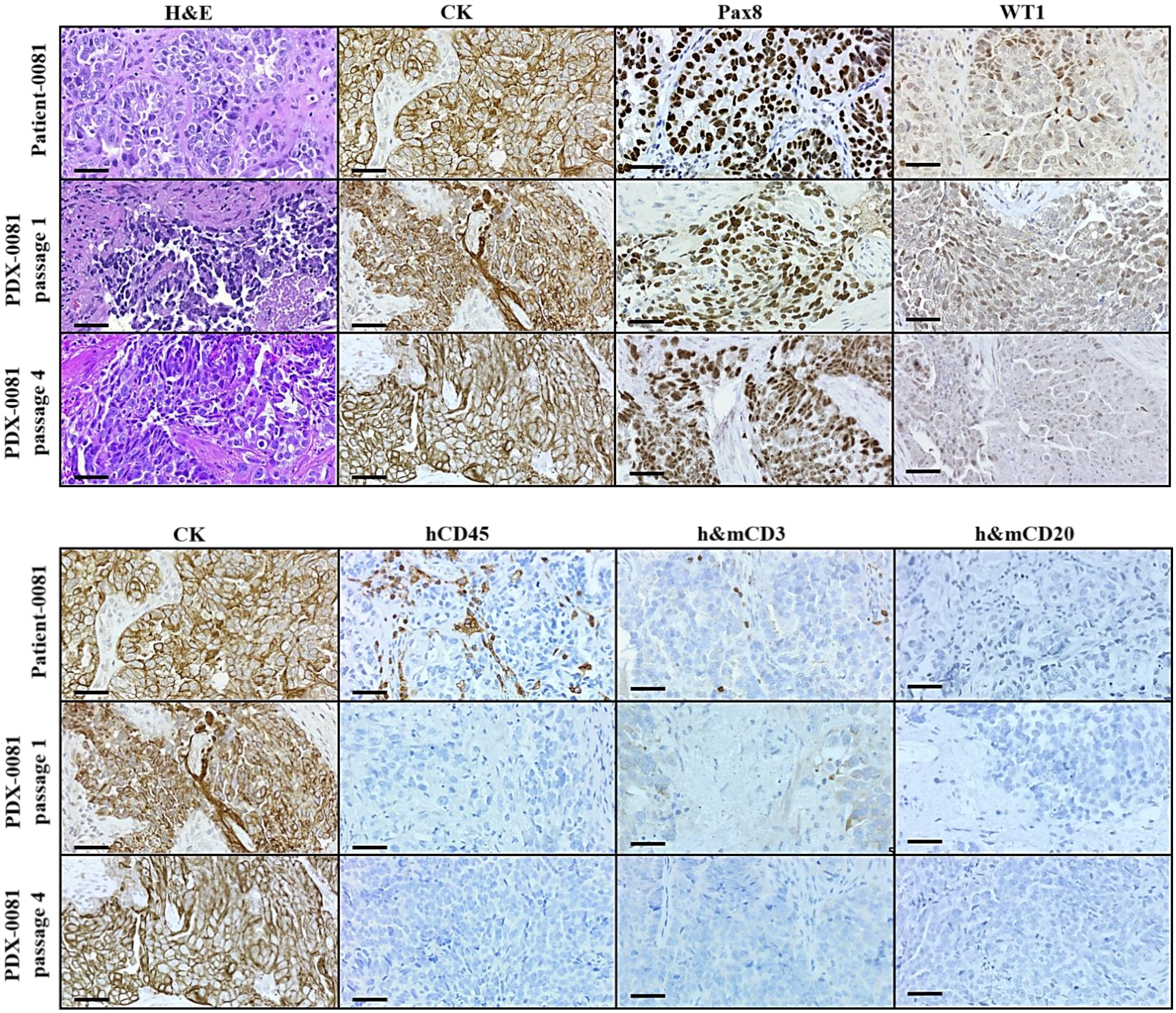
H&E
CK
Pax8
WT1
Patient-0081
PDX-0081 passage 1
PDX-0081 passage 4
CK
hCD45
h&mCD3
h&mCD20
Patient-0081
PDX-0081 passage 1
PDX-0081 passage 4

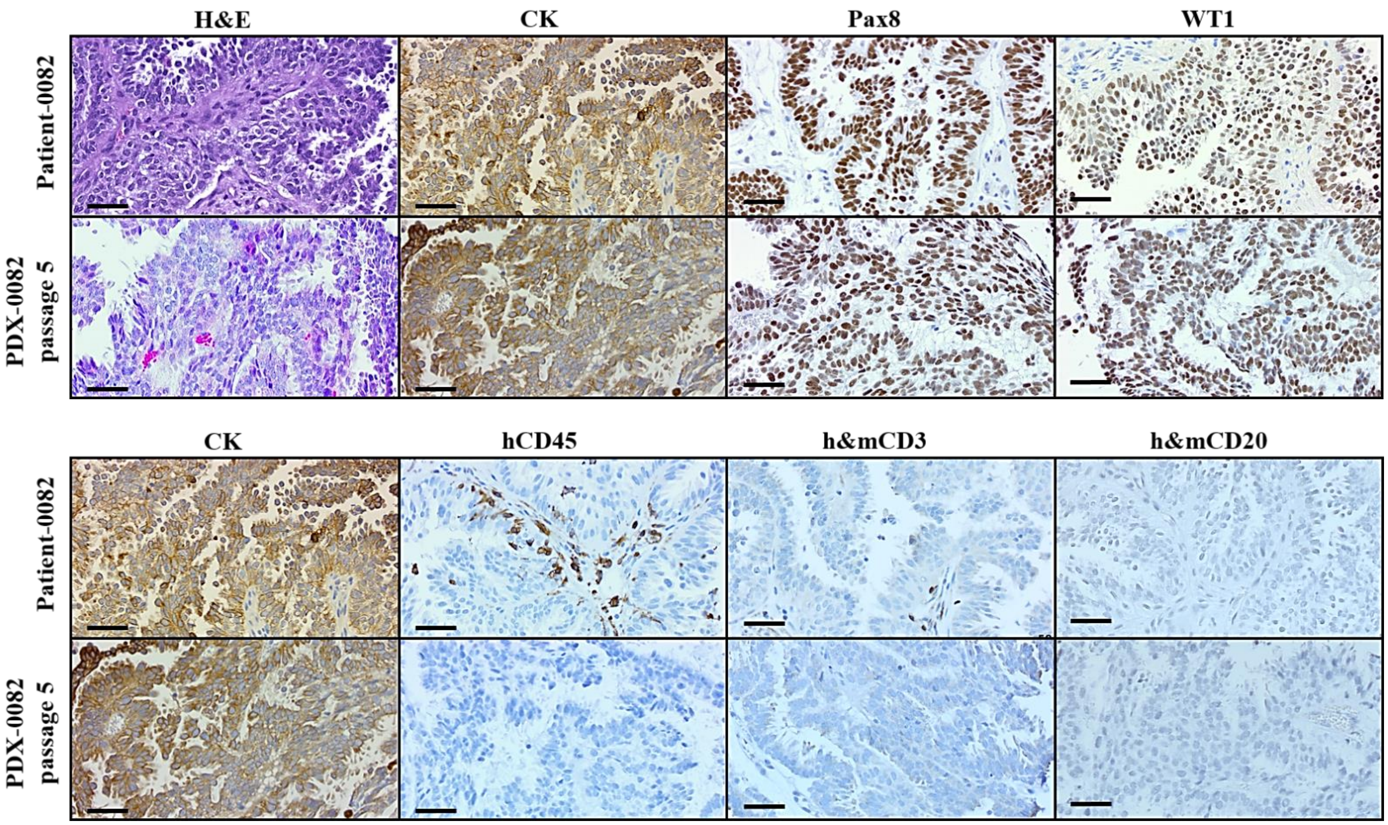
H&E
CK
Pax8
WT1
Patient-0082
PDX-0082 passage 5
CK
hCD45
h&mCD3
h&mCD20
Patient-0082
PDX-0082 passage 5

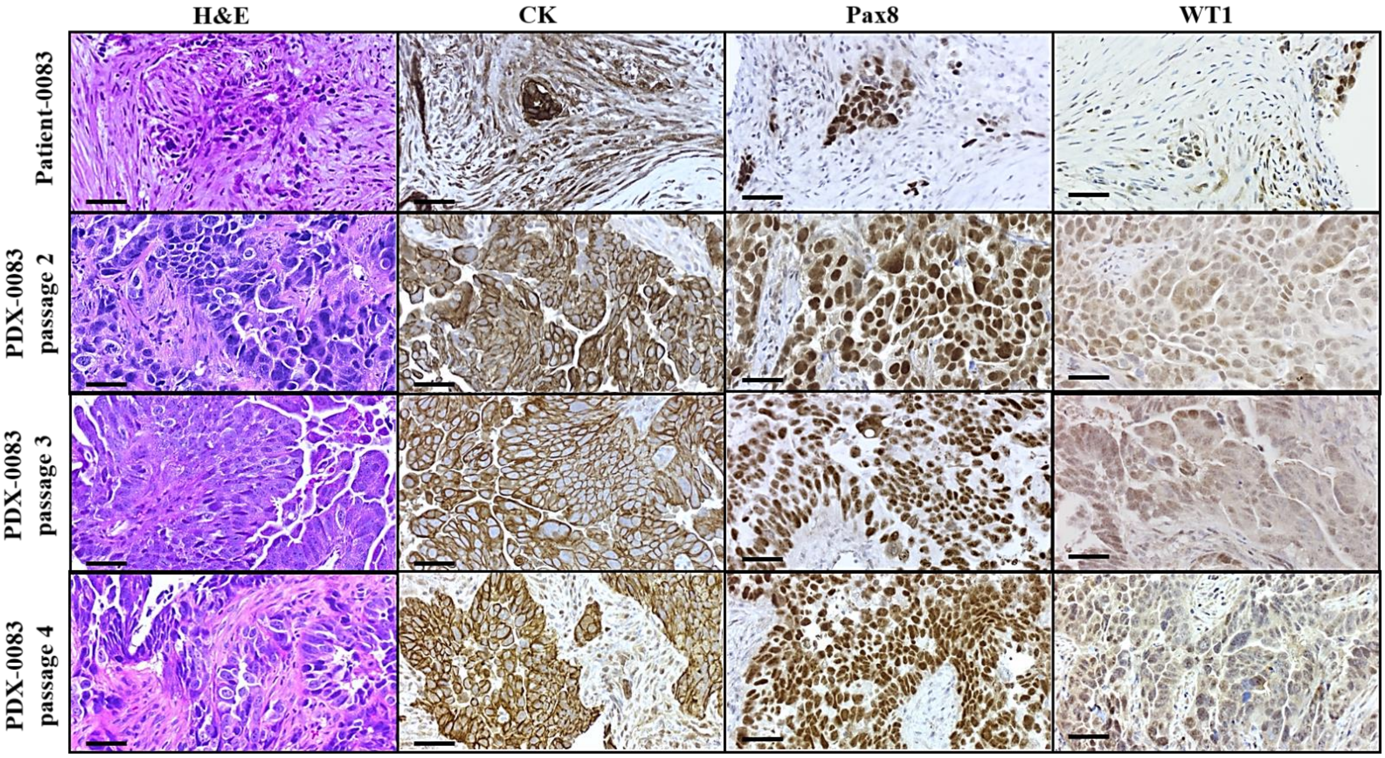

H&E
CK
Pax8
WT1
Patient-0083
PDX-0083 passage 2
PDX-0083 passage 3
PDX-0083 passage 4

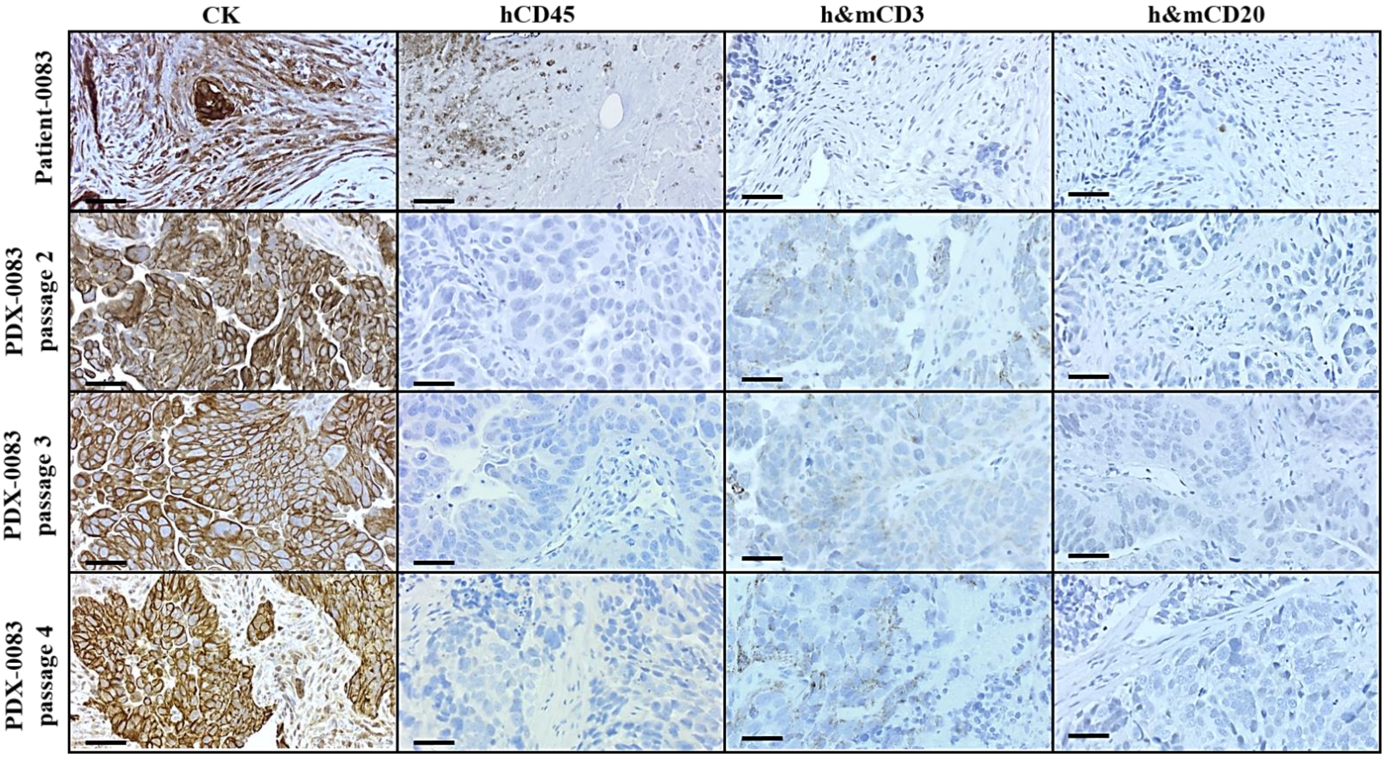

CK
hCD45
h&mCD3
h&mCD20
Patient-0083
PDX-0083 passage 2
PDX-0083 passage 3
PDX-0083 passage 4

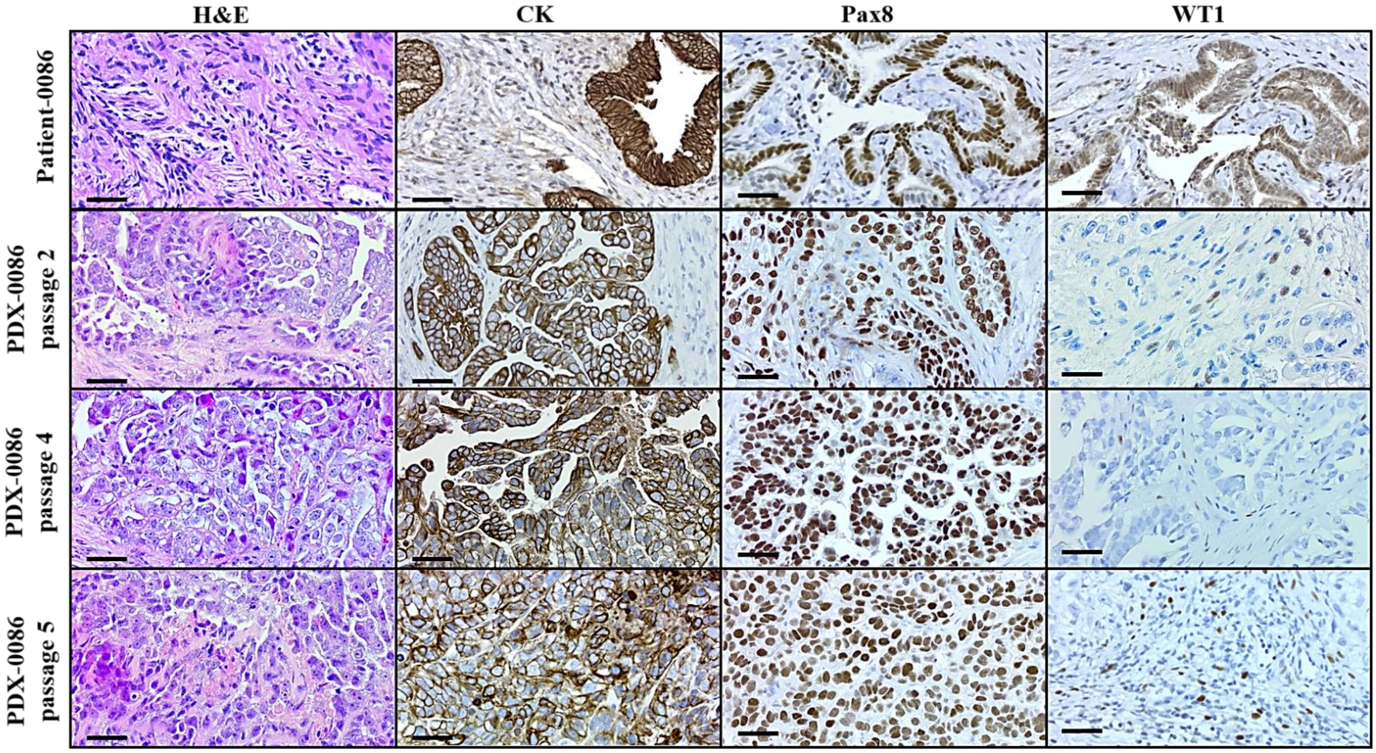
H&E
CK
Pax8
WT1
Patient-0086
PDX-0086 passage 2
PDX-0086 passage 4
PDX-0086 passage 5

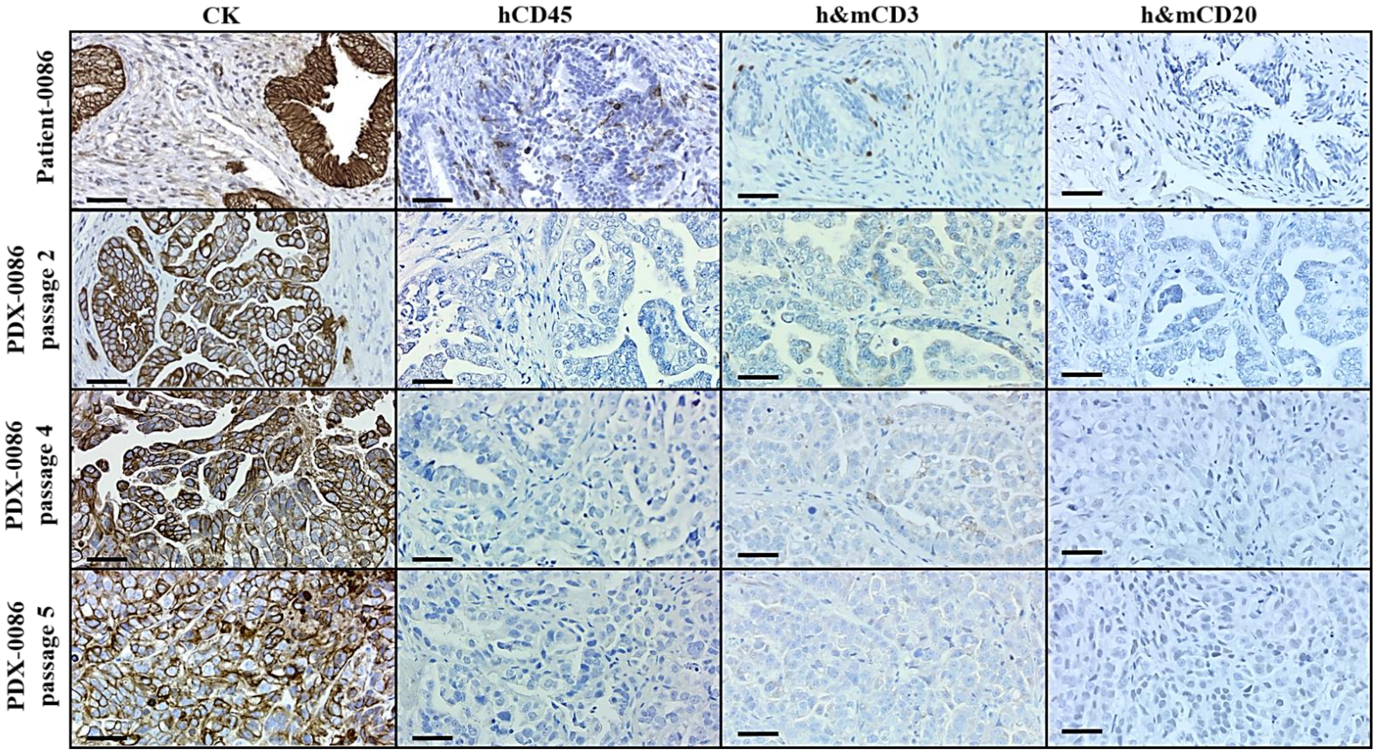
CK
hCD45
h&mCD3
h&mCD20
Patient-0086
PDX-0086 passage 2
PDX-0086 passage 4
PDX-0086 passage 5

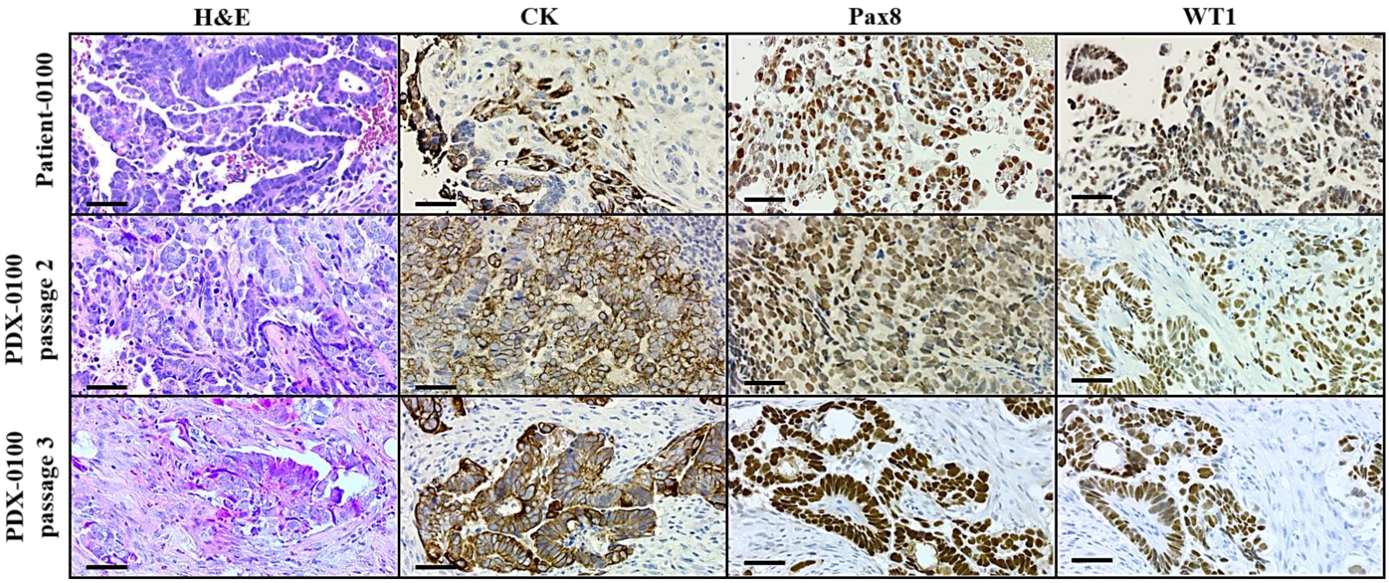
H&E
CK
Pax8
WT1
Patient-0100
PDX-0100 passage 2
PDX-0100 passage 3

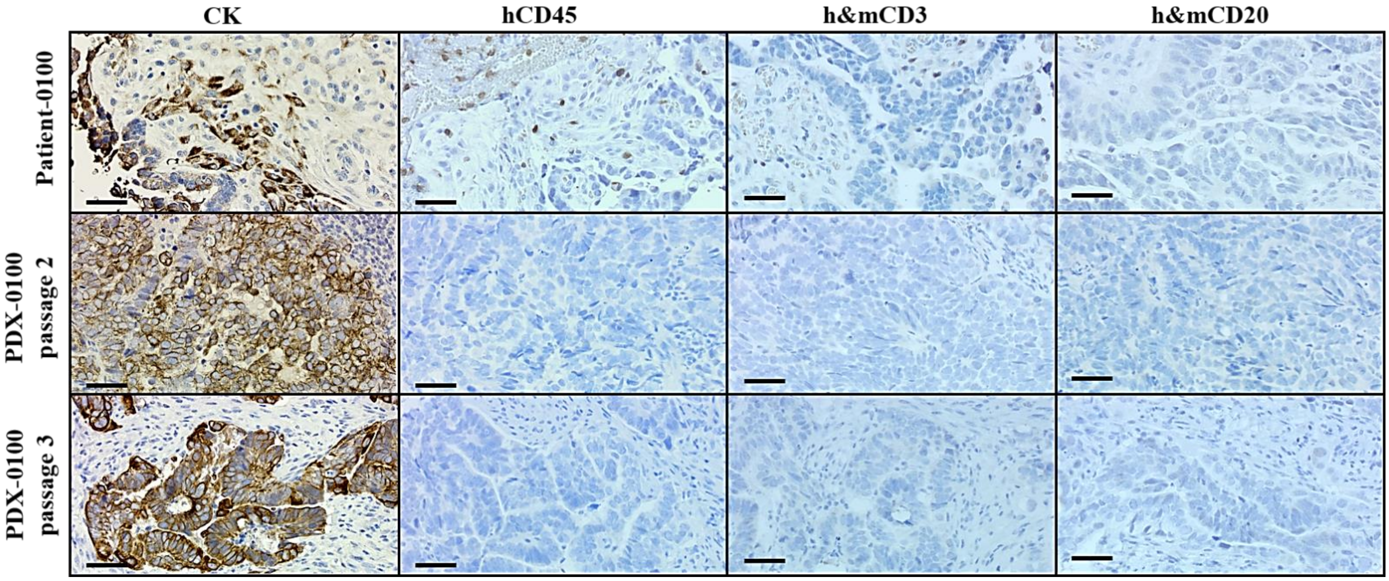
CK
hCD45
h&mCD3
h&mCD20
Patient-0100
PDX-0100 passage 2
PDX-0100 passage 3

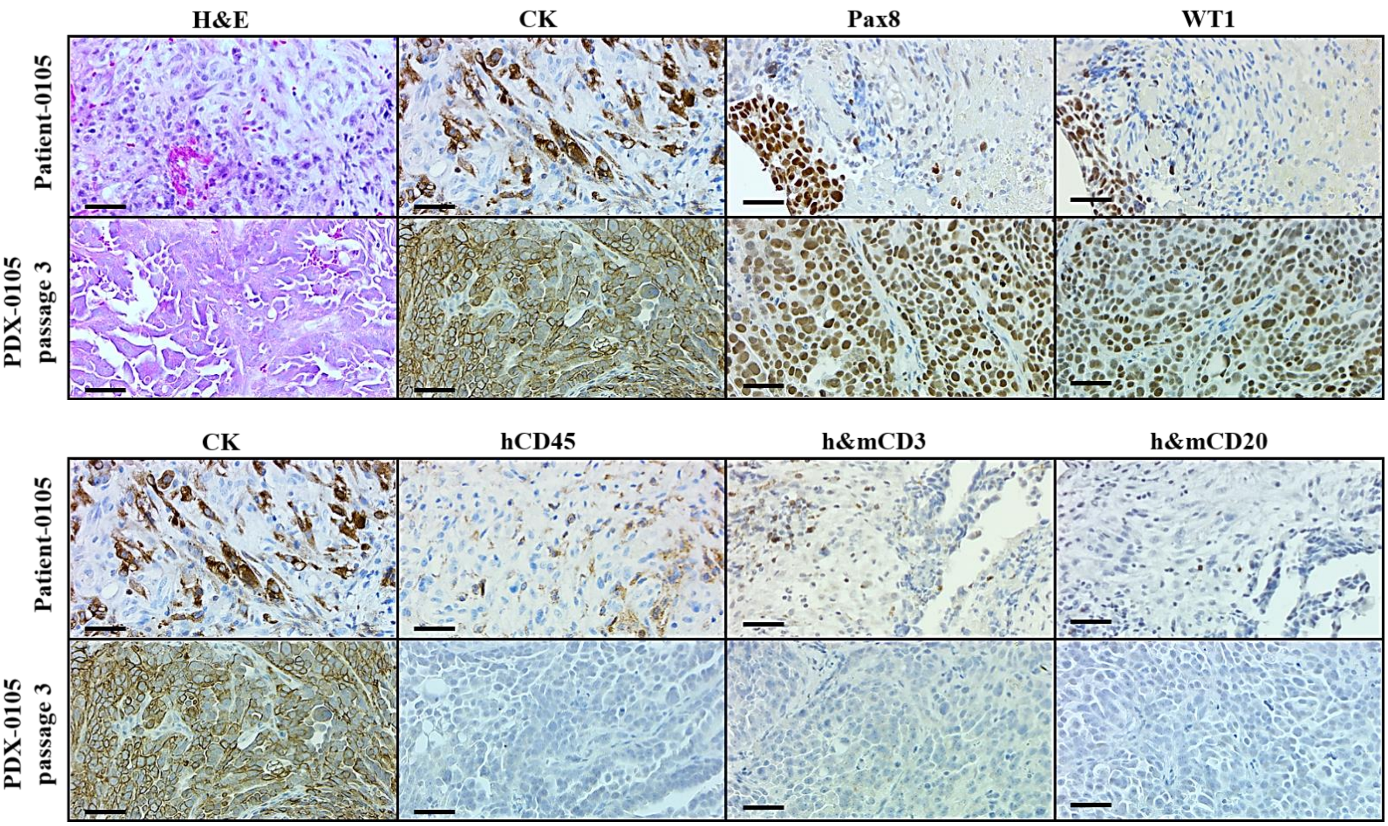
H&E
CK
Pax8
WT1
Patient-0105
PDX-0105 passage 3
CK
hCD45
h&mCD3
h&mCD20
Patient-0105
PDX-0105 passage 3

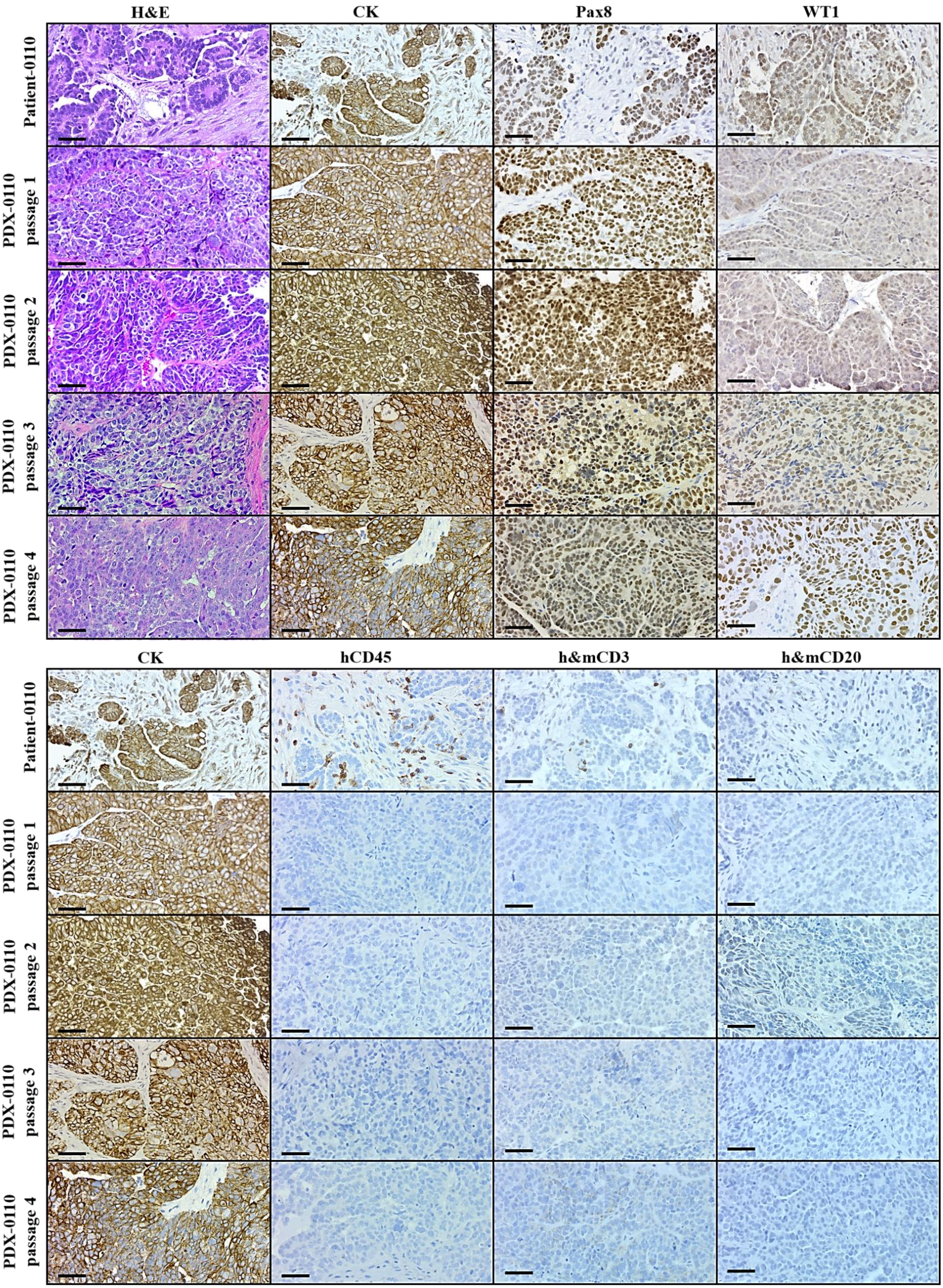
H&E
CK
Pax8
WT1
Patient-0110
PDX-0110 passage 1
PDX-0110 passage 2
PDX-0110 passage 3
PDX-0110 passage 4
CK
hCD45
h&mCD3
h&mCD20
Patient-0110
PDX-0110 passage 1
PDX-0110 passage 2
PDX-0110 passage 3
PDX-0110 passage 4

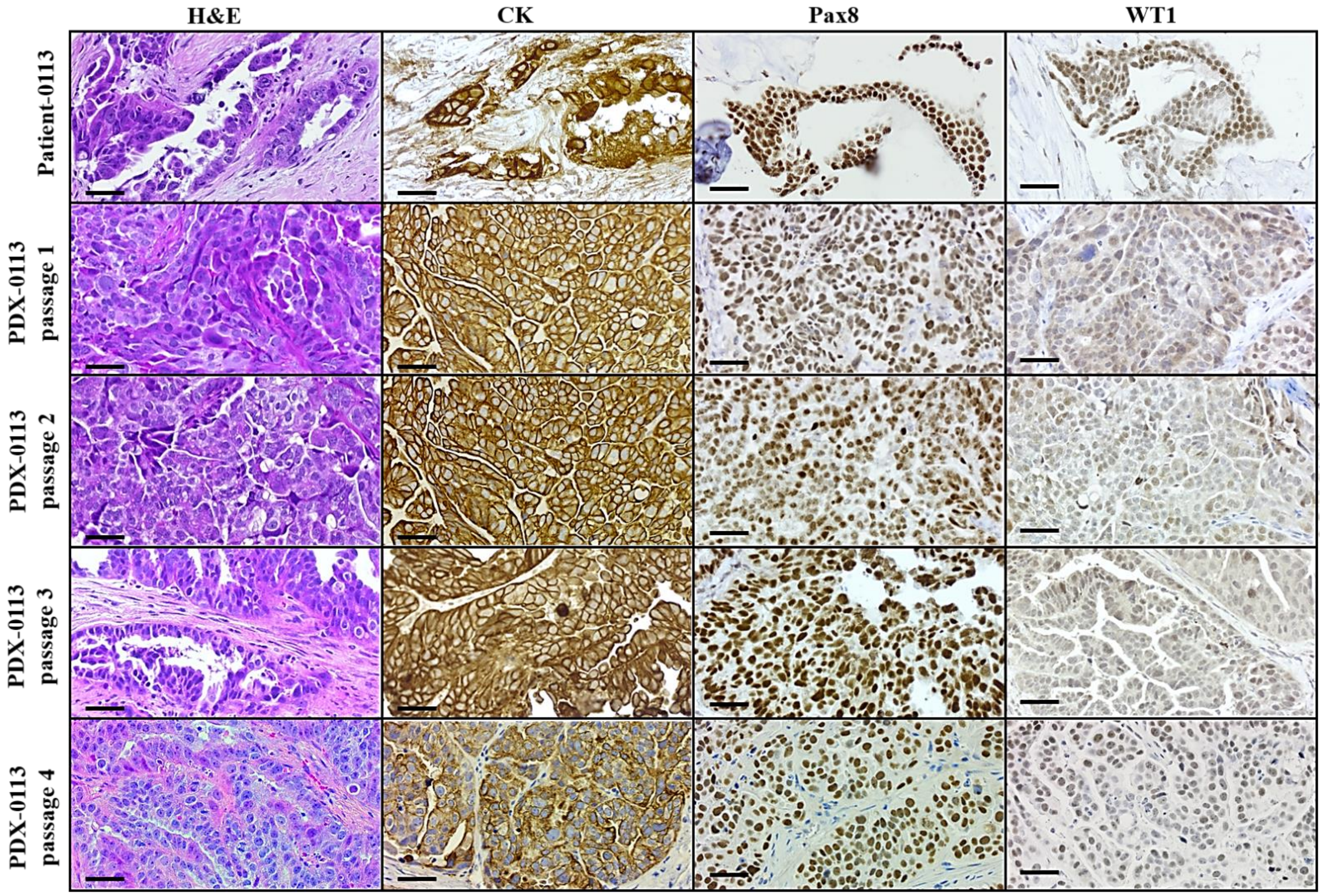
H&E
CK
Pax8
WT1
Patient-0113
PDX-0113 passage 1
PDX-0113 passage 2
PDX-0113 passage 3
PDX-0113 passage 4

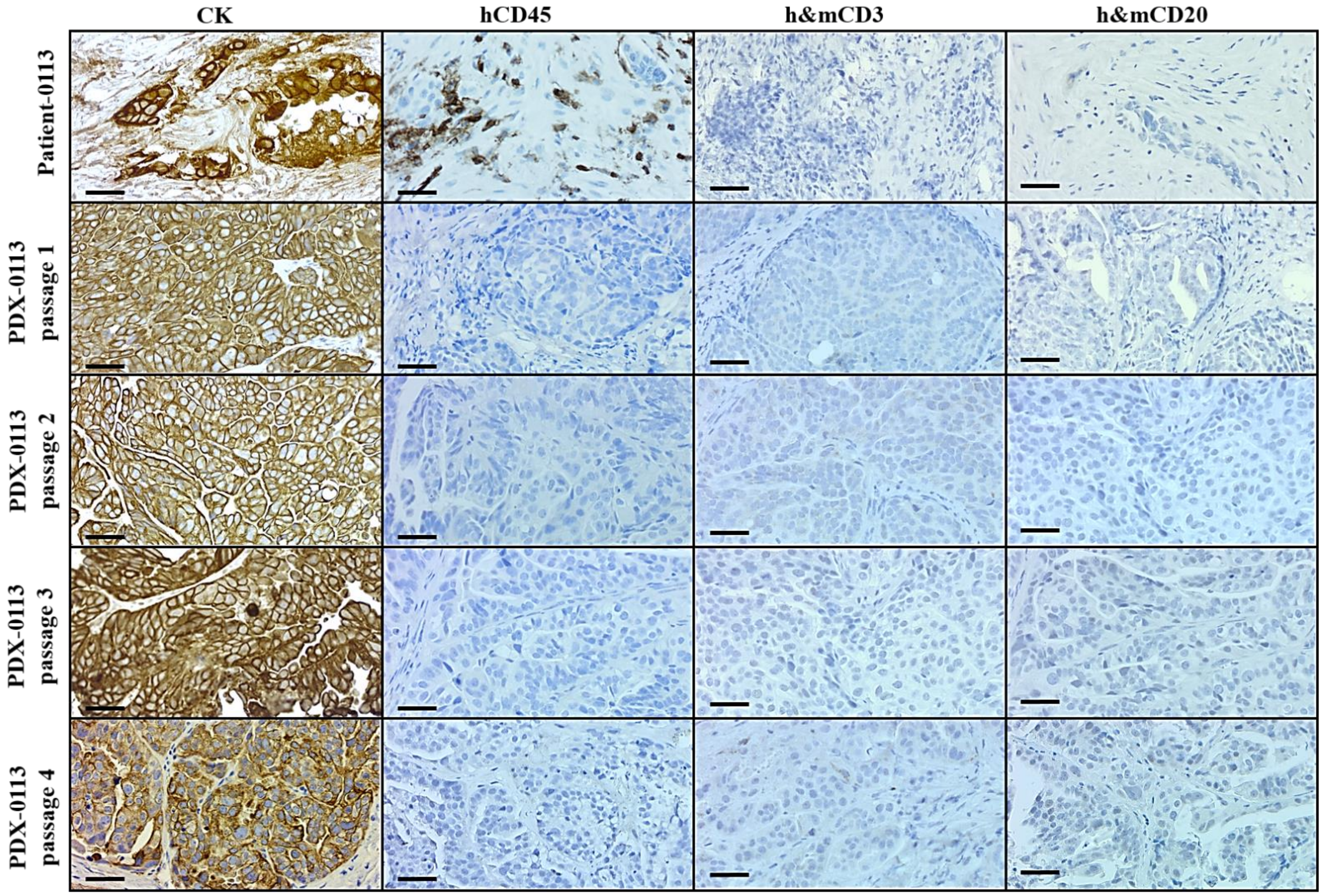
CK
hCD45
h&mCD3
h&mCD20
Patient-0113
PDX-0113 passage 1
PDX-0113 passage 2
PDX-0113 passage 3
PDX-0113 passage 4

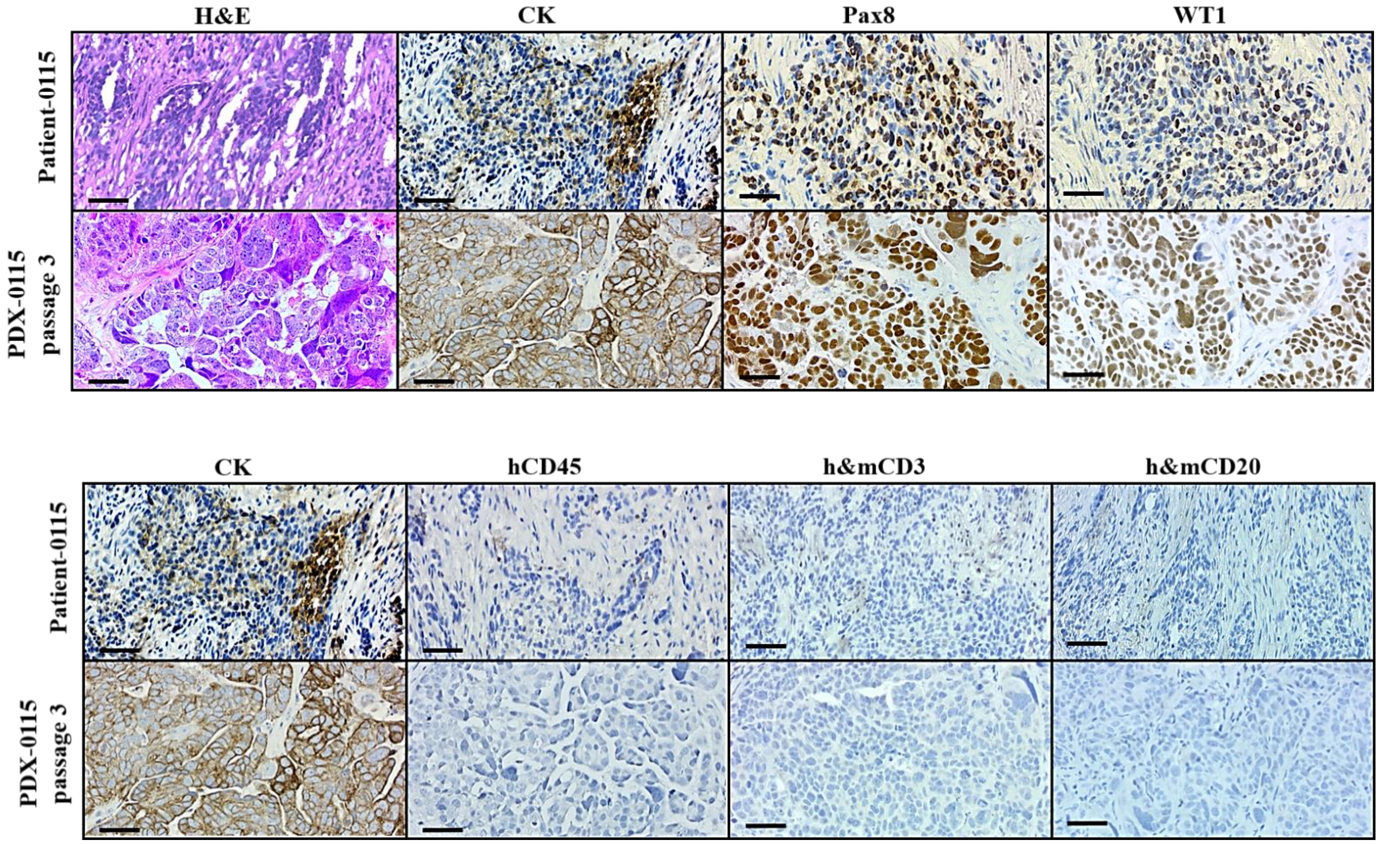

Figure S3. IHC characterization of PDXs. Tumor sections of PDX models across multiple passages were H&E stained and evaluated by IHC for HGSOC subtype markers (CK, PAX8, and WT1) and the presence of human and/or mouse immune cells (hCD45, h&mCD3, and h&mCD20); h – human specific antibody, h&m – human and mouse specific antibody. Scale bars represents 50 µm.

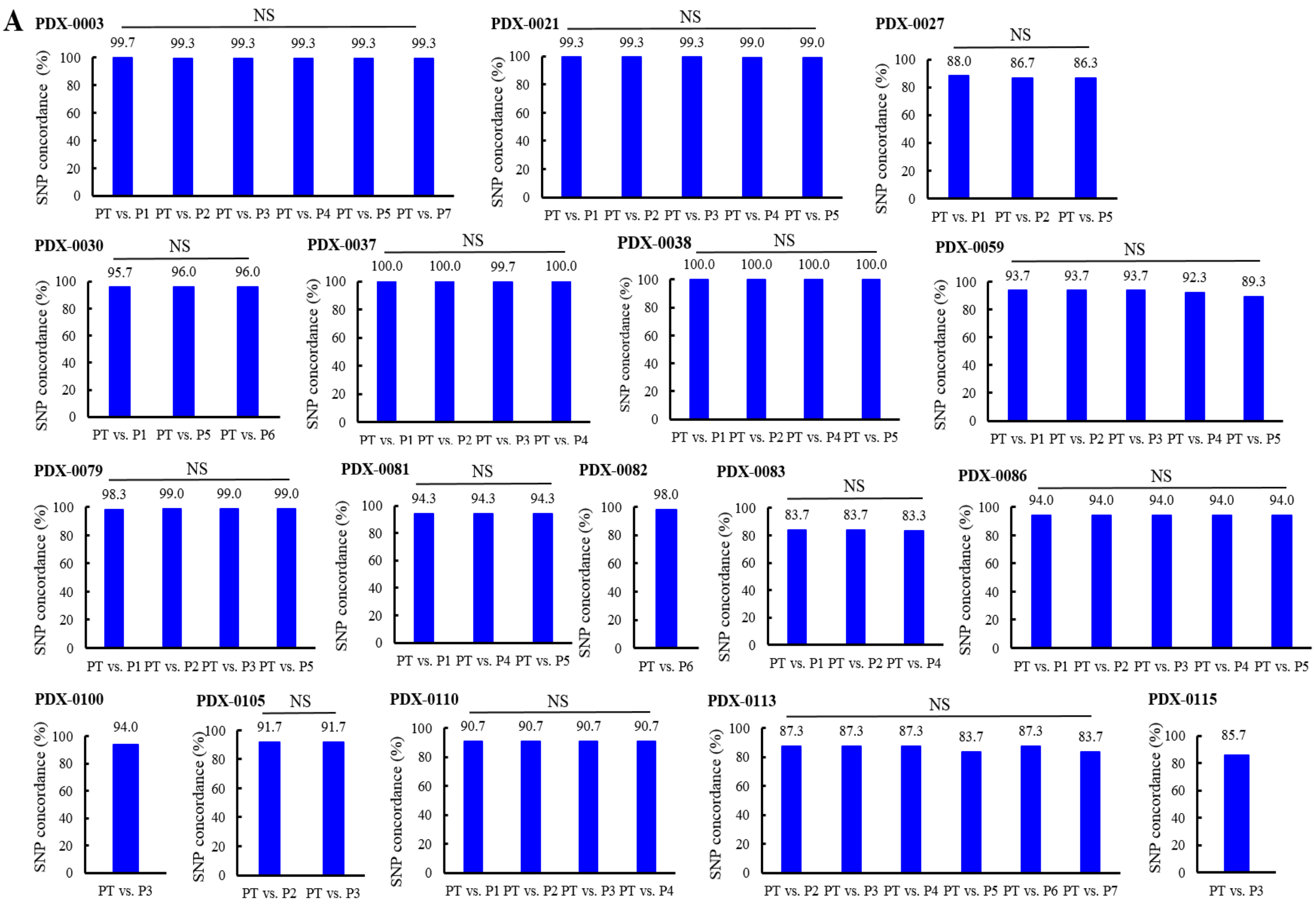
A
PDX-0003
NS
99.7 99.3 99.3 99.3 99.3 99.3
SNP concordance (%)
PT vs. P1 PT vs. P2 PT vs. P3 PT vs. P4 PT vs. P5 PT vs. P7
PDX-0021
NS
99.3 99.3 99.3 99.0 99.0
PT vs. P1 PT vs. P2 PT vs. P3 PT vs. P4 PT vs. P5
PDX-0027
NS
88.0 86.7 86.3
PT vs. P1 PT vs. P2 PT vs. P5
PDX-0030
NS
95.7 96.0 96.0
PT vs. P1 PT vs. P5 PT vs. P6
PDX-0037
NS
100.0 100.0 99.7 100.0
PT vs. P1 PT vs. P2 PT vs. P3 PT vs. P4
PDX-0038
NS
100.0 100.0 100.0 100.0
PT vs. P1 PT vs. P2 PT vs. P4 PT vs. P5
PDX-0059
NS
93.7 93.7 93.7 92.3 89.3
PT vs. P1 PT vs. P2 PT vs. P3 PT vs. P4 PT vs. P5
PDX-0079
NS
98.3 99.0 99.0 99.0
PT vs. P1 PT vs. P2 PT vs. P3 PT vs. P5
PDX-0081
NS
94.3 94.3 94.3
PT vs. P1 PT vs. P4 PT vs. P5
PDX-0082
98.0
PT vs. P6
PDX-0083
NS
83.7 83.7 83.3
PT vs. P1 PT vs. P2 PT vs. P4
PDX-0086
NS
94.0 94.0 94.0 94.0 94.0
PT vs. P1 PT vs. P2 PT vs. P3 PT vs. P4 PT vs. P5
PDX-0100
94.0
PT vs. P3
PDX-0105
NS
91.7 91.7
PT vs. P2 PT vs. P3
PDX-0110
NS
90.7 90.7 90.7 90.7
PT vs. P1 PT vs. P2 PT vs. P3 PT vs. P4
PDX-0113
NS
87.3 87.3 87.3 83.7 87.3 83.7
PT vs. P2 PT vs. P3 PT vs. P4 PT vs. P5 PT vs. P6 PT vs. P7
PDX-0115
85.7
PT vs. P3
0 20 40 60 80 100

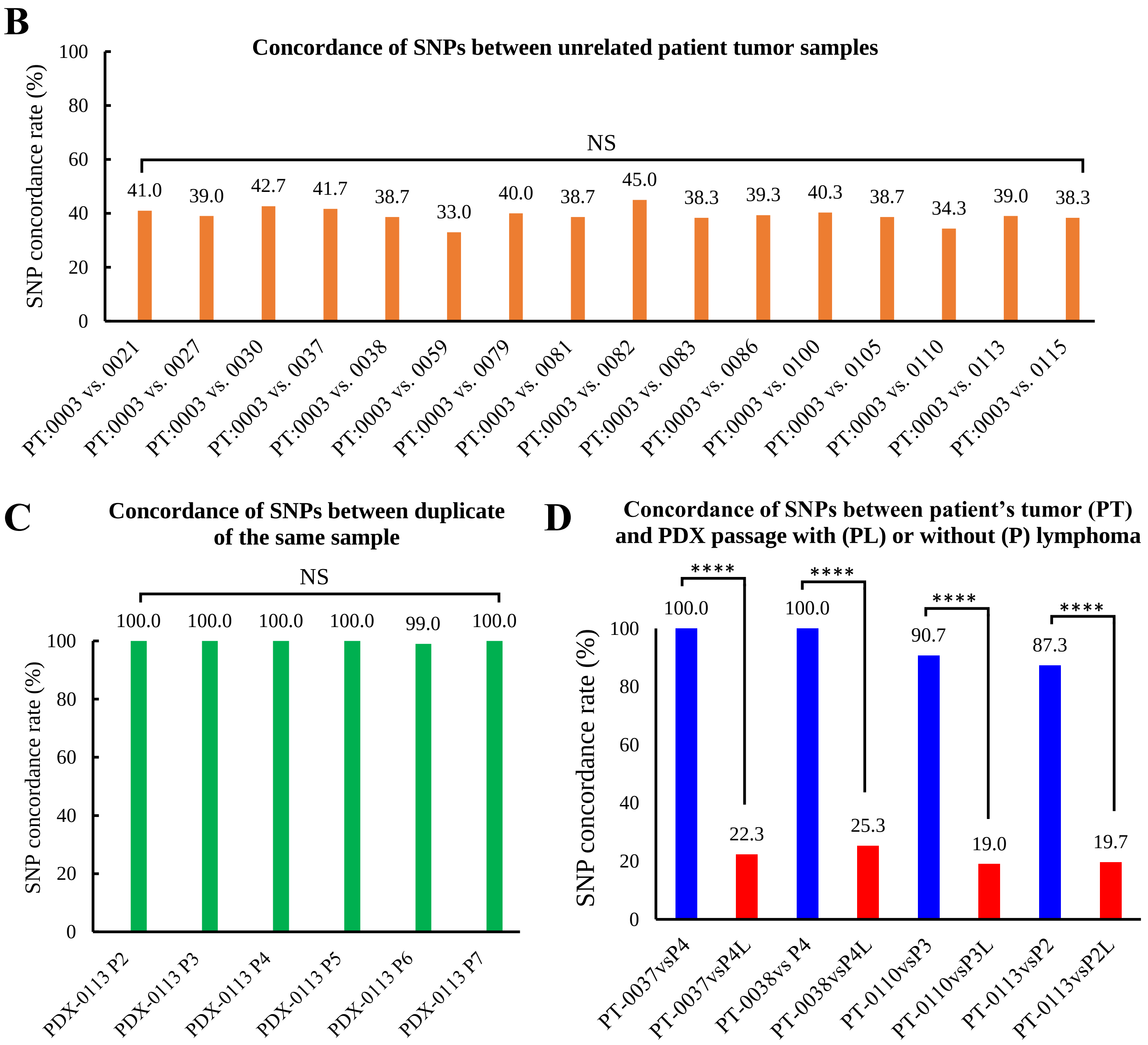

Figure S4. Analysis of molecular landscape and genetic drift of individual ovarian PDX models throughout their derivation and serial propagation. (A) Graphs represent SNP concordance presented as percentage of matching SNPs between original patient tumor and derivative, consecutive PDX passages. Some PDX lines show small continuous genomic evolution through passaging reflected as decrease in SNP concordance that becomes more evident in late PDX passages. (B) Analysis of SNP concordance between unrelated patient's ovarian tumors (PT). This analysis showed that unrelated tumor samples match only on 33.0% - 45.0% of SNPs. (C) Graph represents SNP concordance presented as percentage of matching SNPs between duplicates of different passages of PDX-0113. As expected, this control experiment showed that the duplicates of the same sample match on 100% of SNPs in majority of cases. (D) Graph shows an analysis of SNP concordance between patient's tumor (PT) and respective PDX passage with (PL) or without (P) lymphoma contamination. **A-D.** Statistical significance of data was assessed by one way ANOVA; NS = not significant, and **** = p<0.0001.

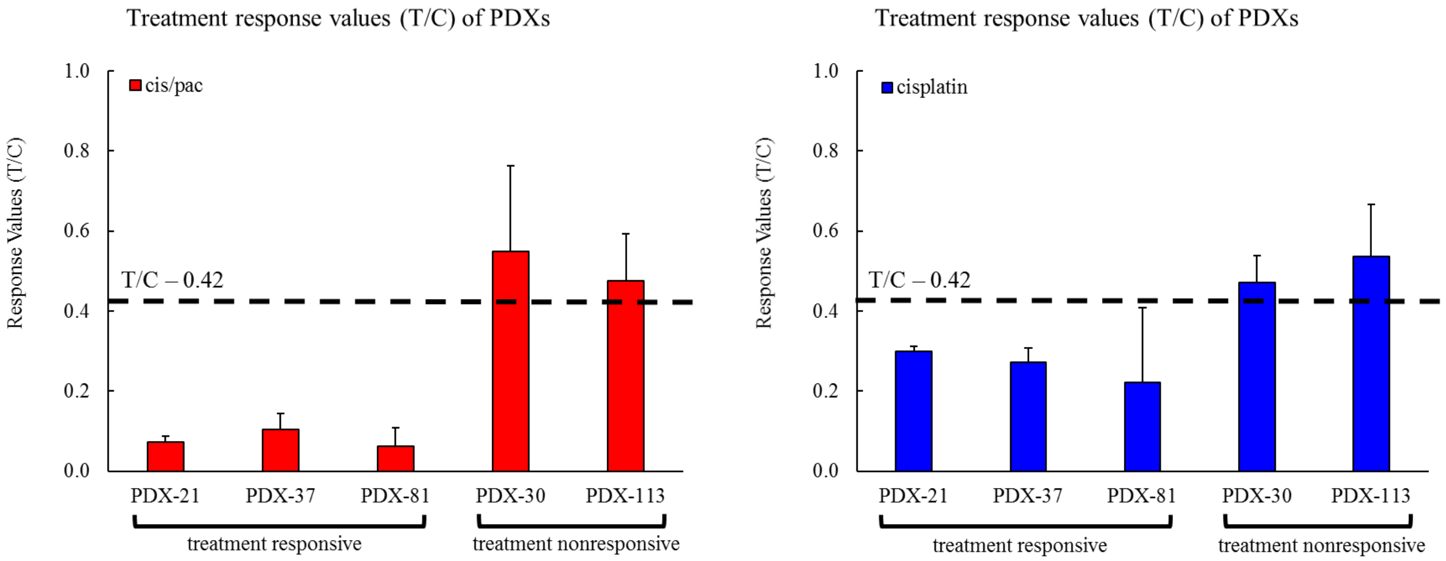

Figure S5. Treatment response values (T/C) of PDX tumor models. The tumor-to-control (T/C) method was used to calculate the ratio between the average volume of the treatment group and the average volume of the control group 6 weeks after treatment began (we selected 6 week time point to calculate T/C across PDXs since control group in some of the PDX models reached endpoint at this time). We adopted a previously described method for treatment response classification based on T/C values (Malcolm et. al.). The treatment response criteria assign the treatment as highly responsive ($T/C<0.1$), responsive (T/C 0.1-0.42), or nonresponsive ($T/C>0.42$). Based on this classification, PDX-0021, PDX-0037 and PDX-0081 are responsive to cisplatin and cisplatin/paclitaxel treatment, while PDX-0030 and PDX-0113 models are nonresponsive to these therapies.

Malcolm JE, Stearns TM, Airhart SD, Graber JH, Bult CJ: Factors that influence response classifications in chemotherapy treated patient-derived xenografts (PDX). PeerJ 2019, 7:e6586.

Table S1. Clinicopathological characteristics of study patients diagnosed with high-grade serous ovarian cancer (HGSOC).

No	PDX ID	Age	Tumor stage	First line treatment	Recurrence (months)	Platinum status	Disease status	OS (months)	BRCA status	Successful engraftment
1	0003	44	IIIC	T/C	4	resistant	DOD	47.8	Negative	YES
2	0010	43	IVB	T/P	11	sensitive	DOD	39.0	*BRCA1*	YES
3	0021	75	IIIC	T/C	8	sensitive	DOD	18.0	N/A	YES
4	0027	66	IIIC	T/C	N/A	sensitive	DOC	12.0	*BRCA2*	YES
5	0029	72	IIIC	T/C	N/A	sensitive	DOD	20.0	N/A	YES
6	0030	63	IIIC	T/C	5	resistant	DOD	14.0	N/A	YES
7	0032	59	IIIC	T/C	7	sensitive	DOD	30.0	Negative	YES
8	0033	76	IIIC	T/P	NA	sensitive	NED	N/A	Negative	YES
9	0037	54	IIIC	T/C	8	sensitive	DOD	44.0	N/A	YES
10	0038	57	IIIC	T/C	0	refractory	DOD	14.0	N/A	YES
11	0039	58	IIIC	T/C	NA	sensitive	DOC	3.0	N/A	YES
12	0054	56	IIIC	T/C	5	resistant	DOD	36.0	Negative	YES
13	0056	61	IIC	T/P	NA	sensitive	NED	N/A	*BRCA2*	NO
14	0059	68	IIIC	T/C	0	resistant	DOD	16.0	Negative	YES
15	0060	46	IVB	T/C	NA	sensitive	NED	N/A	Negative	YES
16	0062	73	IIIC	T/C	23	sensitive	AWD	N/A	Negative	YES
17	0064	58	IIIC	T/C/veliparib	5	resistant	DOD	31.0	Negative	YES
18	0065	54	IIIC	T/C	8	sensitive	DOD	18.0	N/A	YES
19	0067	63	IIIC	T/C	3	resistant	DOD	19.0	Negative	NO
20	0070	71	IVB	T/C	10	sensitive	DOD	17.0	Negative	YES
21	0071	63	IIIC	T/C	3	resistant	DOD	19.0	Negative	NO
22	0073	55	IVB	T/C	5	resistant	DOD	17.0	Negative	NO
23	0075	54	IIIC	T/C	20	sensitive	AWD	N/A	Negative	NO
24	0076	75	IIIC	T/C	7	sensitive	DOD	15.0	Negative	YES
25	0079	71	IIIC	T/C	12	sensitive	DOD	47.0	Negative	YES
26	0081	75	IIIC	T/C	14	sensitive	DOC	14.0	N/A	YES
27	0082	72	IIIC	N/A	N/A	N/A	N/A	N/A	N/A	YES
28	0083	78	IIIC	T/C	4	resistant	DOD	37.5	Negative	YES
29	0086	67	IIIC	T/C	3	resistant	DOD	10.0	N/A	YES
30	0100	61	IVB	T/C	9	sensitive	DOD	25.0	Negative	YES
31	0105	49	IVA	T/C	14	sensitive	DOD	35.0	Negative	YES
32	0110	63	IIIC	T/C	3	resistant	DOD	24.5	Negative	YES
33	0113	58	IIIC	T/C	3	resistant	DOD	19.0	Negative	YES
34	0114	74	IIIC	T/C	19	sensitive	DOD	26.0	Negative	NO
35	0115	55	IIIC	T/C	8	sensitive	DOD	28.0	*BRCA1*	YES
36	0122	63	IVB	T/C	17	sensitive	AWD	N/A	Negative	NO
37	0137	75	IIIC	T/C	7	sensitive	DOD	28.0	N/A	YES
38	0139	65	IIIC	T/C	12	sensitive	AWD	N/A	Negative	NO
39	0152	69	IIIC	T/C	10	sensitive	AWD	N/A	Negative	YES
40	0165	46	IVA	T/C	5	resistant	DOD	32.0	Negative	NO
41	0193	61	IVB	T/C	6	resistant	DOD	27.0	N/A	YES
42	0204	58	IIIC	T/C + BMI1i	6	resistant	AWD	N/A	N/A	YES
43	0236	51	IIIC	T/C	15	sensitive	AWD	N/A	N/A	NO

Abbreviations: T/C, paclitaxel and carboplatin; T/P, paclitaxel and cisplatin; BMIi, inhibitor of BMI1; AWD, alive with disease; NED, no evidence of disease; DOD, died of disease; DOC, died from other cause, OS overall survival, N/A indicates that the respective status is unknown.

BRCA mutations were detected using the Myriad MyRisk testing platform (Myriad genetics); “Negative” result indicates lack of *BRCA* mutations.

Table S2. Summary of engraftment rates of PDXs.

Notes	PDX ID	Successful engraftment	Mouse strain	Tissue Source
Fully characterized PDXs expanded through multiple passages	PDX-0003	YES	NOD/scid	Fresh
	PDX-0021	YES	NRG	Fresh
	PDX-0027	YES	NSG	Fresh
	PDX-0030	YES	NSG	Frozen/thawed
	PDX-0037	YES	NSG	Fresh
	PDX-0038	YES	NSG	Frozen/thawed
	PDX-0059	YES	NRG	Frozen/thawed
	PDX-0079	YES	NRG	Frozen/thawed
	PDX-0081	YES	NRG	Frozen/thawed
	PDX-0082	YES	NRG	Frozen/thawed
	PDX-0083	YES	NRG	Frozen/thawed
	PDX-0086	YES	NRG	Frozen/thawed
	PDX-0100	YES	NOD/scid	Frozen/thawed
	PDX-0105	YES	NOD/scid	Frozen/thawed
	PDX-0110	YES	NOD/scid	Frozen/thawed
	PDX-0113	YES	NOD/scid	Frozen/thawed
	PDX-0115	YES	NOD/scid	Frozen/thawed
[a]Slow-growing PDXs expanded for ~2 passages and cryopreserved	PDX-0029	YES	NSG	Frozen/thawed
	PDX-0039	YES	NSG	Frozen/thawed
	PDX-0054	YES	NSG	Frozen/thawed
	PDX-0060	YES	NRG	Frozen/thawed
	PDX-0064	YES	NRG	Frozen/thawed
	PDX-0065	YES	NRG	Fresh
	PDX-0070	YES	NRG	Frozen/thawed
	PDX-0076	YES	NRG	Frozen/thawed
	PDX-0137	YES	NOD/scid	Frozen/thawed
	PDX-0152	YES	NOD/scid	Fresh
	PDX-0062	YES	NRG	Frozen/thawed
	PDX-0193	YES	NOD/scid	Frozen/thawed
	PDX-0204	YES	NOD/scid	Fresh
	PDX-0010	YES	NOD/scid	Fresh
	PDX-0032	YES	NSG	Frozen/thawed
	PDX-0033	YES	NOD/scid	Frozen/thawed
Primary engraftment failure	PDX-0056	NO	NRG	Frozen/thawed
	PDX-0067	NO	NRG	Frozen/thawed
	PDX-0071	NO	NRG	Frozen/thawed
	PDX-0073	NO	NRG	Frozen/thawed
	PDX-0075	NO	NRG	Frozen/thawed
	PDX-0114	NO	NOD/scid	Frozen/thawed
	PDX-0122	NO	NOD/scid	Frozen/thawed
	PDX-0139	NO	NOD/scid	Frozen/thawed
	PDX-0165	NO	NOD/scid	Fresh
	PDX-0236	NO	NOD/scid	Fresh

[a]Primary tumor specimens grew in mice for at least 2 passages, however due to very slow tumor growth rate these PDXs were not expanded further.
